# MicroRNAs as Epigenetic Biomarkers of Pathogenetic Mechanisms of the Metabolic Syndrome Induced by Antiseizure Medications: Systematic Review

**DOI:** 10.3390/jcm14072432

**Published:** 2025-04-02

**Authors:** Natalia A. Shnayder, Nikolai A. Pekarets, Natalia I. Pekarets, Diana V. Dmitrenko, Violetta V. Grechkina, Marina M. Petrova, Mustafa Al-Zamil, Regina F. Nasyrova

**Affiliations:** 1 Institute of Personalized Psychiatry and Neurology, V.M. Bekhterev National Medical Research Center for Psychiatry and Neurology, 3 Bekhterev St., 192019 St. Petersburg, Russia; pekaretsnick@mail.ru (N.A.P.); grechkina.vv@mail.ru (V.V.G.); regina_nmrcpn@mail.ru (R.F.N.); 2 Shared Core Facilities “Molecular and Cell Technologies”, V.F. Voino-Yasenetsky Krasnoyarsk State Medical University, 1 Partizan Zheleznyak St., 660022 Krasnoyarsk, Russia; mart2802@yandex.ru (D.V.D.); stk99@yandex.ru (M.M.P.); 3 Department of Psychiatry and Clinical Psychology, Irkutsk State Medical University, 1 Krasny Vosstaniya St., 664003 Irkutsk, Russia; menatalis999@gmail.com; 4 Department of Physiotherapy, Faculty of Continuing Medical Education, Peoples’ Friendship University of Russia, 117198 Moscow, Russia; alzamil@mail.ru; 5 Department of Psychiatry, General and Clinical Psychology, Tula State University, 92 Lenin Ave., 300012 Tula, Russia

**Keywords:** epilepsy, epilepsy treatment, antiepileptic drug, antiseizure medication, adverse drug reaction, metabolic syndrome, biomarker, epigenetic, metabolomic

## Abstract

Antiseizure medication (ASM) induced metabolic syndrome (AIMetS) is a common adverse drug reaction (ADR) of pharmacotherapy for epilepsy and psychiatric disorders. However, the sensitivity and specificity of blood biomarkers may be insufficient due to the influence of combined pathology, concomitant diseases, and the peculiarities of the metabolism of ASMs in patients with epilepsy. **Methods**: The presented results of experimental and clinical studies of microRNAs (miRs) as epigenetic biomarkers of MetS and AIMetS, which were entered into the different databases, were analyzed for the last decade (2014–2024). **Results**: A systematic review demonstrated that miRs can act as promising epigenetic biomarkers of key AIMetS domains. However, the results of the review demonstrated the variable role of various miRs and their paralogs in the pathogenesis of AIMetS. Therefore, as part of this study, an miRs signature was proposed that allows us to assess the risk of developing and the severity of AIMetS as low risk, medium risk, and high risk. **Conclusions**: The mechanisms of development and biomarkers of AIMetS are an actual problem of epileptology, which is still far from being resolved. The development of panels (signatures) of epigenetic biomarkers of this widespread ADR may help to increase the safety of pharmacotherapy of epilepsy. However, to increase the sensitivity and specificity of circulating miRs in the blood as biomarkers of AIMetS, it is necessary to conduct “bridge” studies in order to replicate the results of preclinical and clinical studies into real clinical practice.

## 1. Introduction

Historically, medications used in the treatment of epilepsy have been referred to by a variety of terms, such as “antiepileptic”, “anticonvulsant”, or “antiseizure”. Terminology is important because using terms that do not accurately reflect the action of specific treatments may result in misunderstanding their effects and in inappropriate use. In 2020, the International League against Epilepsy agreed that these drugs should be combined under the common name “antiseizure medication” (ASMs). This term accurately reflects their primarily symptomatic effect against seizures and reduces the possibility of healthcare practitioners, patients, or caregivers having undue expectations or an incorrect understanding of the real action of these medications. The term “antiseizure” to describe these agents does not exclude the possibility of beneficial effects on the course of the disease and comorbidities that result from downstream effects of seizures, whenever these effects can be explained solely by suppression of seizure activity [1,2,3]. Patients with epilepsy take ASMs for a long time, sometimes throughout their lives [4], which increases the risk of adverse drug reactions (ADRs) from various organs and systems [5]. Metabolic syndrome (MetS) is a group of metabolic risk factors including abdominal obesity, high blood pressure, hyperglycemia, triglyceridemia, and decreased serum high-density lipoproteins. According to experts from the International Diabetes Federation (IDF), about 20–25% of the world’s adult population suffers from MetS [6]. An increase in the prevalence of MetS in many countries contributes to an increase in mortality rates [7] and the economic burden of the disease [8]. ASMs-induced metabolic syndrome (AIMetS) is an ADR that occurs in 20–35% of patients with epilepsy and leads to negative consequences, including increased risk of cardiovascular disease, type 2 diabetes, and premature death [9,10,11]. However, AIMetS is the least studied ADR because most of the previous studies examined individual domains of AIMetS (weight gain [12], abdominal obesity [9], hypertension [13], type 2 diabetes [14], etc.) but not AIMetS as a whole [9,10,13].

It is known that the risk of AIMetS is highest in patients taking the first generation of ASMs, including carbamazepine [15], phenytoin [16], valproic acid [13,15], and phenobarbital [17]. The role of new-generation ASMs in the development of AIMetS has not yet been sufficiently studied, and their studies are isolated and have contradictory results [11,18]. This explains the increased interest of researchers and clinicians in the AIMetS problem and the search for biomarkers of this ADR, including clinical biomarkers, metabolic biomarkers [13], and genetic and epigenetic biomarkers [19,20].

**Figure 1:**
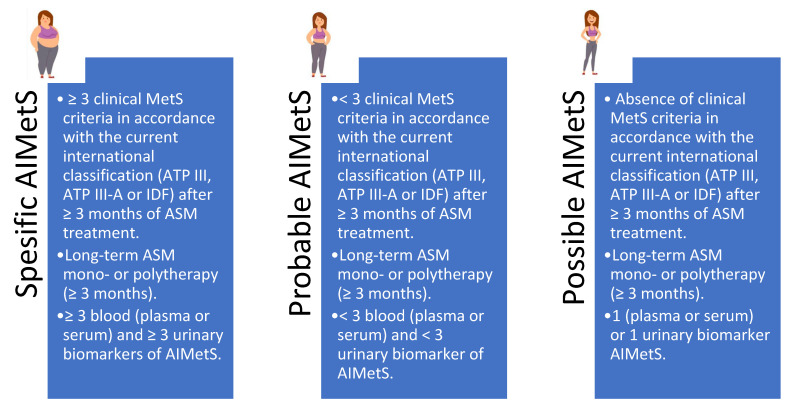
Antiseizure medication induced metabolic syndrome criteria ([13] by authors’ modification). Note: ASM—antiseizure medication; MetS—metabolic syndrome; AIMetS—antiseizure medication induced metabolic syndrome; IDF—the International Diabetes Federation; ATPIII—the Adult Treatment Panel, III edition.

A biomarker is a characteristic that allows an objective assessment of the risk of developing pathological conditions in a particular patient (person). They can be used not only to classify and assess the risk of developing and progressing diseases but also to assess the safety and risk of classical and new therapeutic strategies [21,22] in epileptology, including the risk of developing AIMetS [9,10,11,12,13,14,15,16,17,18]. Thus, based on the additive contribution of clinical and metabolic biomarkers (Table A1), it was proposed to diagnose three variants of AIMetS: specific, probable, and possible [13] (Figure 1).

The epigenetic biomarkers of AIMetS continue to be studied [23,24,25,26]. Candidates for the epigenetic biomarkers of MetS and AIMetS are selected as a result of a large number of fundamental and clinical studies from a huge number of molecules produced by cells and tissues. Epigenetic markers have great potential as clinical tools for diagnosing, predicting, and monitoring the development of AIMetS and facilitating clinical decision making. An epigenetic biomarker is defined as any epigenetic label or altered epigenetic mechanism that does the following:

1. measured in liquids (blood, saliva, and urine) or primary types of tissue specimen (fresh, frozen, and fixed with formalin and filled with paraffin);
2. helps to identify (diagnose) diseases;
3. predicts the severity and outcome of the disease;
4. responds to drug and non-drug therapy;
5. monitors the response of organs and tissues to non-drug or drug therapy;
6. predicts the risk of developing the disease in the future;
7. allows simultaneous diagnosis and targeted therapy (teragnosis) [27].

Epigenetic biomarkers have some advantages over genetic biomarkers because they can provide important information about the function of genes in individual cell types; they can reflect information about the effects of the patient’s environment and lifestyle and medication, thereby explaining, for example, how ASMs, and metabolic factors affect the course of epilepsy and the patient’s overall health; they can provide information about the natural and aggravated history of the disease (epilepsy), as they are real bioarchives; and they are extremely stable in liquids (plasma, saliva, urine, semen, vaginal secretions, breast milk, and cerebrospinal fluid) and very stable in the main types of tissue specimen (fresh and frozen tissues, dried blood stains (Guthrie cards), and paraffin blocks fixed with formalin [27].

The sensitivity and specificity of metabolic (biochemical and hormonal) biomarkers of AIMetS [13] can vary in a wide range depending on the influence of environmental factors (for example, climatic, geographical, cultural, and nutritional) as well as on the age and gender of patients with epilepsy. In addition, the sensitivity and specificity of AIMetS metabolic biomarkers may be influenced by the conditions of sampling and storage of samples. This encourages researchers to search for new biomarkers of AIMetS that would have better stability profile in samples as well as good reproducibility of research results in various laboratories.

Short (19–25 nucleotides) non-coding single-stranded regulatory ribonucleic acids (RNAs)—microRNAs (miRs)—are prospective epigenetic biomarkers that act as post-transcriptional repressors of gene expression. MiRs block messenger RNA (mRNA) by repressing translation or promoting mRNA degradation. This process has been called RNA interference, and it leads to “gene silencing”. MiRs are formed primarily from intronic DNA regions and are found in various organs and tissues of the body. MiRs capable of carrying information through biological fluids (bloodstream, urine, saliva, cerebrospinal fluid, milk, lacrimal, and seminal fluids) are called circulating miRs [28,29,30,31,32,33].

Each person has a genome, but there are many epigenomes depending on the type of cells, which are influenced by environmental factors, including climatic and geographical living conditions, dietary habits, medications taken, and other xenobiotics. In general, epigenetic biomarkers are labels that determine which part of the human genome will be disabled or enabled during certain periods of normal and pathological life, particularly in patients with MetS and AIMetS. Epigenetic biomarkers do not depend on changes in the DNA sequence [34], but they affect the cellular and physiological phenotypic features of an organism or may even be the cause of disease development. Such biomarkers do not include causal gene mutations and genetic variations (for example, single nucleotide polymorphisms) but are associated with functionally significant changes in the human genome due to changes in the regulation of gene expression. Epigenetic biomarkers can be stored during cell division throughout their entire life.

The purpose of this review is to present and systematize the results of experimental and clinical studies of the role of miRs as promising epigenetic biomarkers of a high risk of developing AIMetS.

## 2. Materials and Methods

The results of experimental and clinical studies of microRNAs as epigenetic biomarkers of MetS and AIMetS, which were entered into the databases Google Scholar, PubMed, Scopus, and eLIBRARY.RU, were analyzed for the last decade (2014–2024).

**Figure 2:**
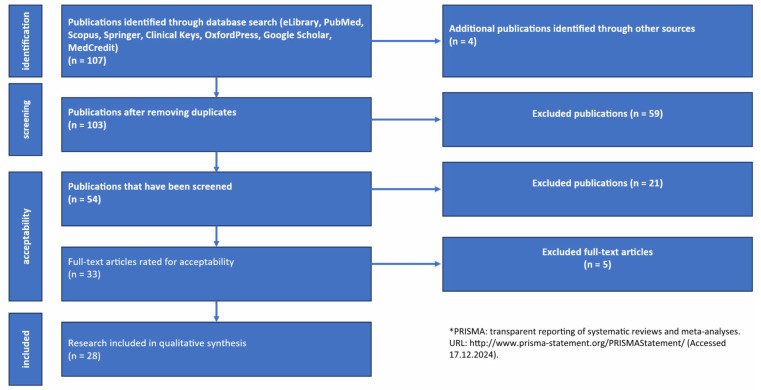
PRISMA flow diagram. Note: *—Preferred Reporting Items for Systematic Reviews and Meta-Analyses.

This review was carried out in accordance with the recommendations of Preferred Reporting Items for Systematic Reviews and Meta-Analysis (PRISMA 2020) [35] (Figure 2). Inclusion criteria: access type—open access to the full-text version of the publication in Russian or English; publication type—original article, systematic review, meta-analysis, or Cochrane review. Exclusion criteria: duplicate publications, posters, conference materials, dissertations, and abstracts of dissertations published as manuscripts (Table 1).

The search terms included keywords and their combinations: “Epilepsy”; “Metabolic Syndrome”; “Obesity”; “Dyslipidemia”; “Hyperglycemia”; “Microrna”; “Antiepileptic Drugs/Antiseizure Drugs”; “Systemic Inflammation”; “Oxidative Stress”; “Atherogenesis”; “Appetite”.

Changes in the expression level of the analyzed miRs were evaluated in biological fluids (blood, plasma, serum, exosomes, and mononuclears) of patients and in animal models as biomarkers of the development of MetS or AIMetS as well as in the main mechanisms of the development of MetS or AIMetS.

## 3. Results

### 3.1. Epigenetic Biomarkers of ASM-Induced Metabolic Syndrome

**Figure 3:**
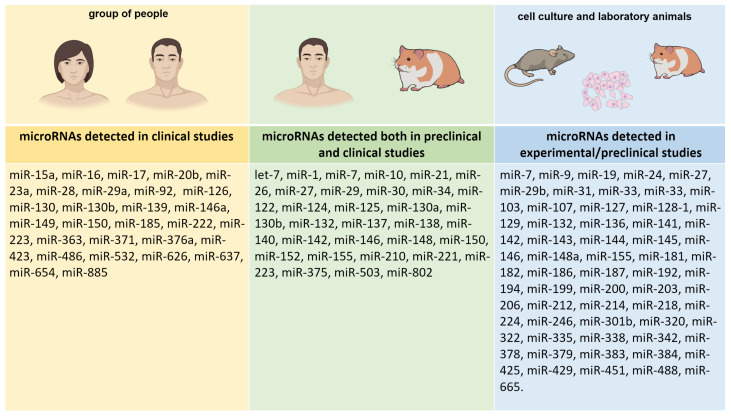
List of microRNAs depending on the type of study. Note: Clinical studies n = 6; experimental/preclinical studies n = 22.

Since small interfering RNAs and miRs are closely related, MiRs can also play an important role in controlling DNA methylation and histone modifications; also, miRs can regulate the chromatin structure by acting on key histone modifiers [36]. Thus, miRs can be considered important participants in the epigenetic control of gene expression. They are known to be involved in the regulation of various physiological and pathological processes that play an important role in the mechanisms of the development of MetS and AIMetS, including oxidative stress [37,38,39], systemic inflammation [40,41], adipocyte differentiation and central obesity [40,42,43], lipid and glucose metabolism [40,43,44,45,46,47,48,49,50,51,52,53,54,55,56], regulation of appetite [55,57,58,59,60], changes in neuropeptide Y (NPY) expression [55,61], changes in leptin responsiveness [40,60], changes in orexin expression [62], changes in testosterone levels [63], thyroid hormones [56], and parathyroid hormone [64] (Table 2). The studies were both clinical and preclinical, using cell cultures and animal models (Figure 3).

### 3.2. MiRs as Epigenetic Biomarkers of the Main Domains of ASM-Induced Metabolic Syndrome

#### 3.2.1. Oxidative Stress

There are numerous clinical and experimental reports demonstrating ASM-induced changes in the oxidant–antioxidant balance in patients with epilepsy. ASMs of the first (phenobarbital) and second (phenytoin and valproic acid)-generation activate lipid peroxidation and the formation of reactive oxygen species in patients with epilepsy, which induces an increase in the levels of pro-oxidative enzymes (superoxide dismutase and catalase) while lowering the levels of protective enzymes (glutathione peroxidase and glutathione reductase) [65].

Compared to first-generation ASMs, second-generation ASMs (levetiracetam and lamotrigine) reduce lipid peroxidation and reactive oxygen species levels, while oxcarbazepine significantly reduces markers of oxidative stress in adult patients with epilepsy [66,67].

An increase in reactive oxygen species levels affects the expression of a number of microRNAs that have both pro-oxidative and cytoprotective effects. Some microRNAs can inhibit oxidative stress by modulating the Nrf2 signaling pathway and have a high degree of control over this pathway at different stages of its development. NFE2L2/Nrf2 (nuclear factor 2, related to erythroid factor 2) is a critical biomarker associated with cytoprotective reactions in response to oxidative and electrophilic disorders. Nrf2 is ubiquitinated and directed to degradation by the Kelp-like ECH-associated protein 1 (Keap1). The Keap1/Nrf2 pathway is the most important cytoprotective pathway responding to increased levels of reactive oxygen species [68]. MiRs are involved in the control of the Nrf2 pathway through several mechanisms: changing the nuclear translocation of Nrf2; influencing the expression of Nrf2; control of upstream Nrf2 mediators; and Keap1 modulation. Thus, miR-24 initiates the Nrf2 signaling pathway by inhibiting Keap1 [37] and exerts antioxidant and cytoprotective effects.

Sirtuin 1 (Sirt1) is a NAD+-dependent deacetylase, which is an important antioxidant enzyme associated with the development of diabetes mellitus. Sirt1 uses the first pathway, PGC1a/Era, to induce Nrf2, although it is also able to directly activate Nrf2 using other mechanisms. For example, patients taking valproic acid had markedly lower plasma levels of Sirt1 than healthy volunteers [69]. Much less studied is Sirt3 [70], which positively regulates Forkhead Box Protein O1 and O2, which leads to an antioxidant response [38]. Sirt3 is expressed in tissues and organs with a high metabolic rate, including the liver, brain, heart, and brown adipose tissue, and it plays an important role in the regulation of oxidative stress and mitochondrial metabolism [71], therefore, the effect of microRNAs on the expression level of this molecule is of undoubted scientific and clinical interest.

Increased expression of miR-99a can directly suppress the expression of NAD(P)H-oxidase 4, thereby reducing the level of oxidative stress [38,72]. In turn, by favorably influencing the Nrf2 signaling pathway, miR-125b prevents oxidative damage to cells by regulating *PRXL2A* [38].

MiR-128-3p reduces the damaging effect of oxidative stress by increasing the levels of Sirt1, Nrf2, Sirt3, NAD(P)H quinonedehydrogenase 1, and hemoxidase (HO-1). Focusing on the F1-Nrf2-HO-1 axis, miR-141 suppresses the migration and proliferation of vascular smooth muscle cells, preventing local oxidative reactions in inflammatory diseases such as atherosclerosis [37].

By targeting Keap1 [73] and suppressing Nrf2 signals, miR-152 protects cells from oxidative stress. MiR-200a can inhibit oxidative stress by degrading Keap1 and activating the expression of the Nrf2 antioxidant pathway, while miR-455 stimulates the activation of Nrf2 through a higher-level mediator histone deacetylase 2 [37].

By destroying cullin 3, which, in turn, activates genes associated with the antioxidant response, such as *NQO1, HO-1,* and *GCLC*, miR-455 stabilizes and increases the level of Nrf2. To protect cells from oxidative damage, miR-626 activates Nrf2 and modulates the effects of Keap1 [37].

The mechanisms of oxidative stress induction are also related to the Nrf2 signaling pathway. Thus, miR-23b induces oxidative stress by decreasing the expression of Sirt1 and Nrf2 [38,74].

Overexpression of miR-34a suppresses the expression of the gene encoding Nrf2, with a marked decrease in the levels of mRNA and Nrf2 protein. At the same time, when miR-34a expression is suppressed, the Nrf2-dependent antioxidant pathway is restored [38,75].

MiR-92a inhibits Sirt1 and endothelial nitric oxide synthase and also activates inflammasomes, which exacerbates endothelial dysfunction under oxidative stress [38].

MiR-128 reduces the expression level of the Sirt1 protein and the expression level of the Nrf2 protein, contributing to the development of oxidative stress [37,38]. MiR-140 works insignificantly at the Nrf2 and Sirt2 levels, replacing the expression levels of HO-1, NAD(P)H Quinonedehydrogenase 1, GCML, Keap1 and Forkhead Box Protein O3a [37].

MiR-27a, miR-142, miR-144, and miR-153 are regulatory microRNAs for Nrf2, reducing their transcription [37]. The activation and expression of the Nrf2 signaling pathway and its downstream mediator HO-1 are markedly accelerated when the expression of miR-320 is suppressed [37], and miR-383 mediates oxidative stress and apoptosis, suppressing peroxiredoxin 3 [38].

#### 3.2.2. Systemic Inflammation

ASMs affect the synthesis of pro-inflammatory cytokines in a complex way. Valproates inhibit production of tumor necrosis factor alpha (TNF-α) and interleukin 6 (IL-6) in vitro. However, valproates increase IL-1, IL-6, and IL-5 levels in patients with epilepsy. Carbamazepine stimulates the production of IL-10, IL-2, and transforming growth factor-β (TGF-β) in vitro, while phenytoin increases the level of IL-1 and the release of TGF-β [76,77].

However, the results of studies of the relationship between taking ASMs and changes in the levels of biomarkers of the systemic inflammatory response are contradictory and demonstrate both pro-inflammatory and anti-inflammatory effects, so their role in the development of AIMetS remains controversial [78,79].

Systemic inflammation is characterized by increased serum levels of acute phase proteins and proinflammatory cytokines, including C-reactive protein, TNF-α, IL-1b, IL-6, and IL-17 as well as infiltration by macrophages and T-lymphocytes in insulin-dependent tissues [39]. In general, miRs regulate various stages of inflammation, starting with the initiation, expansion, and resolution of both positive and negative inverse connection. With positive inverse connection, a set of events limits not only the invasion of pathogens but also the successful restoration of damaged tissues. On the contrary, negative inverse connection activated in severe inflammation helps maintain tissue homeostasis.

MiRs have been identified in recent years that are capable of inhibiting systemic inflammation through their altered expression in certain immune cells. At the same time, a number of miRs have proinflammatory effects. As part of the inflammatory response, miR biogenesis is often regulated at various stages, such as synthesis, processing, and stabilization of pre- or mature miRs [39,40].

MiR-7 suppresses the growth and development of vascular smooth muscle cells stimulated by platelet growth factor-BB by targeting epithelial growth factor [40]. MiR-9 inhibits the formation of inflammasomes and suppresses the systemic inflammatory response by targeting the Janus kinase 1/signal converter and transcription activator 1 pathway in macrophages [40,80].

MiR-10a is a central regulator of the universal transcription factor NF-ĸB signaling pathway. Dysregulation of NF-ĸB induces and supports systemic inflammation, which is one of the key links in epileptogenesis [81]. Diseases associated with systemic inflammation are characterized by overexpression of miR-10a, and overexpression of two key targets of miR-10a, i.e., mitogen-activated protein kinase and a protein containing β-transducin repeats, is accompanied by activation of the proinflammatory signaling pathway NF-ĸB and hyperproduction of proinflammatory molecules (IL-6, IL-8, MCP-1, and VCAM-1) [40].

MiR-124 is also known to mediate the cholinergic anti-inflammatory response by targeting the signal converter and transcription activator 3 and TNF-α-converting enzyme, thereby limiting the secretion of pro-inflammatory cytokines (IL-6 and TNF-α) by macrophages [40]. MiR-125b enhances its anti-inflammatory potential by altering mitogen-activated protein kinase signaling mediated by TNF receptor-associated factor-6 (TRAF6) and NF-ĸB, thus regulating IL-1β gene expression [40].

MiR-126, another endothelial microRNA, inhibits vascular inflammation and targets Sprouty-related, EVH1 domain-containing protein 1, regulatory subunit 2 of phosphoinoside kinase type 3 (PI3KR2) [82], and the vascular cell adhesion molecule type 1 (VCAM-1). The anti-inflammatory effect of miR-142 is associated with the regulation of IL-1 receptor-associated kinase and inhibition of the production of proinflammatory cytokines (NF-ĸB1, TNF-α, and IL-6) [40].

MiR-143 suppresses the regulation of the TLR4/MyD88/NF-ĸB pathway, thereby exerting an anti-inflammatory effect [40]. MiR-149 has an anti-inflammatory effect by modulating TAK1/NF-ĸB signaling, and miR-181a suppresses TNF-α [40].

Deficiency of miR-146a leads to increased expression of inflammatory cytokines IL-1β and IL-18. The anti-inflammatory mechanism of miR-223 is to inhibit the classical proinflammatory reaction of M1 macrophages by suppressing the signaling of toll-like receptor-4 (TLR4) [39,40,83].

Pro-inflammatory miRs are also associated with regulation of NF-ĸB and inflammatory mediators. Elevated levels of let-7a have been found to activate the NF-ĸB proinflammatory pathway by targeting the nuclear factor inhibitor ĸB kinase. Increased levels of miR-21 in adipose tissue in people with obesity and type 2 diabetes, which is essentially the leading clinical manifestation of MetS associated with sluggish systemic inflammation and increased adipogenic differentiation through modulation of TGF-β1 signaling. MiR-21 also plays a crucial role in angiogenesis through regulation of vascular endothelial growth factor A [40,84,85].

The proinflammatory mechanism of miR-23a is associated with the activation of the proinflammatory pathway NF-ĸB in M1 macrophages, with simultaneous inhibition of the anti-inflammatory Janus kinase 1/signal converter and transcription activator 6 pathway [40,80].

Deficiency of the antioxidant Sirt1 induces miR-132 expression in primary human preadipocytes, which induces the release of proinflammatory cytokines.

It has been established that the miR-34 family (miR-34a and miR-34c) induces the release of proinflammatory cytokines and chemokines by targeting G protein LGR4, thereby delaying the local inflammatory response [37,86].

Overexpression of proinflammatory miR-92a leads to increased regulation of several genes encoding proinflammatory cytokines in recipient macrophages [40], and overexpression of miR-138 and miR-155 promotes systemic inflammation by activating the proinflammatory signaling pathway NF-ĸB as well as the MyD88 and TRIF pathways, which contribute to increased levels of proinflammatory cytokines and suppression of the cytoprotective protein Sirt1 [40,87].

MiR-200 demonstrates a pro-inflammatory response by its effect on the synthesis of the protein Zeb-1, which is involved in increasing the activity of the cyclooxygenase-2 and monocytic chemotactic protein-1 in vascular smooth muscle cells in type 2 diabetes [40]. The Zeb-1-neuroinflammation axis plays an important role both in epileptogenesis and in the development of MetS in patients with epilepsy and epileptic syndromes receiving ASMs [88,89].

#### 3.2.3. Regulation of Adipogenesis and Development of Central Obesity

Adipogenesis is the process by which adipocytes develop from fatty stem cells to form adipose tissue [90]. Impaired adipogenesis and regulation of adipose tissue metabolism are associated with various domains of AIMetS (obesity, type 2 diabetes, and cardiovascular diseases). Understanding the secretome of adipocytes is important for the development of targeted therapy for MetS and AIMetS in children and adults with epilepsy and epileptic syndromes [91].

Older-generation ASMs (phenytoin and valproic acid) can induce adipogenesis, which is accompanied by the overexpression of the gene encoding the gamma-activated peroxisome proliferator (*PPARγ*) receptor [14]. In addition, carbamazepine enhances the differentiation of 3T3-L1 preadipocytes into mature adipocytes by increasing the expression of PPARy, CCAAT/enhancer-binding protein β (C/EBPβ), and fatty acid synthase in 3T3-L1 preadipocytes while suppressing the signals of the Wnt/β-catenin pathway [92].

MiRs can have a direct regulatory effect on the regulation of adipogenesis. Studies have established an inhibitory effect on adipogenesis and the development of central obesity in a number of miRs. One of the mechanisms of microRNA-mediated regulation of adipogenesis is the regulation of the expression of TGF-β, a protein that regulates adipocyte proliferation, differentiation, and growth and modulates the expression and activation of other growth factors, including IF-γ and TNF-α [91].

Ectopic expression of intergenic miR-27a in 3T3-L1 preadipocytes suppresses adipocyte differentiation by reducing PPARγ expression [41]. The loss of miR-33 family has a profound negative effect on eating behavior in rodents and leads to abnormally high food consumption, followed by the development of obesity and insulin resistance [42].

Wnt/β-catenin signaling is a negative regulator of adipogenesis [92]. Thus, miR-27, miR-181, and miR-344 inhibit adipogenesis by activating Wnt signaling in 3T3-L1 preadipocytes [41].

MiR-138 and miR-182 inhibit the expression of both key adipogenic transcription factors C/EBPα and PPARγ [41,43].

MiR-145 is a potent inhibitor of adipogenesis, reducing the activity of the PI3K/Akt and mitogen-activated protein kinase signaling pathways [93], and miR-146a-5p inhibits TNF-α-induced adipogenesis by targeting insulin receptors in primary adipocytes [41].

MiR-363 inhibits adipocyte differentiation by activating the gene encoding transcription factor *E2F2* and suppressing the expression of C/EBPα and PPARγ [39]. Air-448 inhibits adipocyte differentiation by translational repression of krupel-like factor 5 [41].

MiRs also induce adipogenesis and central obesity. Thus, miR-21 stimulates adipogenesis by modulating the signaling of TGF-β1 preadipocytes. Increased adipogenic differentiation in 3T3-L1 preadipocytes is associated with increased expression of miR-17, miR-21a, miR-21, and miR-143, which stimulate the expression of adipogenic transcription factor C/EBPα and enhance the transmission of TGF-β signals [39,41].

MiR-103 doubles the expression of adipogenic transcription factor PPARγ, also increasing the expression of fatty acid binding protein 4 and adiponectin by about nine and four times, respectively [41]. MiR-128-1 regulates the homeostasis of circulating lipoproteins as well as the expression of genes encoding PPAR and other regulators of fatty acid oxidation and systemic inflammation, leading to the development of central obesity [42].

MiR-144 reduces Forkhead Box Protein O1 expression by suppressing its stimulating effect on adiponectin, thereby weakening the inhibitory effect of adiponectin on adipogenesis in 3T3-L1 preadipocytes, and miR-146b promotes adipogenesis in 3T3-L1 preadipocytes by suppressing Sirt1 expression [41].

Some miRs (for example, miR-148a, miR194, miR-210, and miR-322) induce adipogenesis by suppressing Wnt pathway signaling [41]. Hyperexpression of miR-375 initiates adipocyte differentiation by increasing C/EBPα and PPARγ levels and simultaneously inducing fatty acid binding protein 4 in adipocytes and triglyceride accumulation. Hyperexpression of miR-375 promotes adipogenesis through differentiation of 3T3-L1 preadipocytes and suppression of signal-regulated extracellular protein kinase ERK1/2 phosphorylation [41].

#### 3.2.4. Changes in Lipid Metabolism

Various mechanisms of the dysregulation of lipid metabolism caused by taking ASMs of various generations are being actively studied [94,95], including ASM-induced hypercholesterolemia and an increased risk of atherosclerosis in patients with epilepsy [13].

MiRs have a direct effect on lipid metabolism [42]. They mediate the liver’s response to metabolic stress associated with lipid overload by suppressing the expression of the nuclear transcription factor of hepatocytes 4A, which is a critical transcription factor in lipid metabolism. MiR-223 is another regulatory center that suppresses genes involved in the biosynthesis of cholesterol. In addition, miR-223 suppresses the expression of the SR-BI receptor absorber by hepatocytes, which transports cholesterol from HDL to the liver. The final result of the action of MiR-223 is a decrease in the level of cholesterol in the liver [42].

MiR-246b affects the mRNA of the β-receptor of thyroid hormone in the liver, which leads to changes in the expression of genes responsible for lipid metabolism and a decrease in the content of intracellular lipids [56].

The mechanisms of action of miR-30c, miR-33a, miR-33b, miR-128-1, miR-144, and miR-148a are described below since the main point of application is the regulation of LDL and HDL cholesterol homeostasis [42,44,45,46].

MiR-7 activates the expression of sterol regulatory element-binding proteins 1 and 2, which are transcription factors and activate gene expression to enhance lipid uptake and synthesis of fatty acids (sterols) in hepatocytes [42,96]. MiR-27a regulates lipid metabolism in the liver, so its role has been most studied using the example of non-alcoholic fatty liver disease (NAFLD). In addition, miR-27b acts as a regulatory operator controlling the networks of the lipid metabolism genome [42].

MiR-122 regulates various genes involved in the synthesis of cholesterol and fatty acids in the liver and is also discussed in more detail when discussing NAFLD [42].

#### 3.2.5. Changes in High-Density Lipoprotein Cholesterol Homeostasis

MiR–33a and miR-33b coordinate inhibition of the reverse transport of cholesterol from the periphery back to the liver by suppressing ATP-binding cassette transporters A1 and also inhibit the removal of cholesterol from the body by suppressing the protein transporter ATP-binding cassette transporters B11 and lipid-transporting ATPase 8B1, which promotes the removal of cholesterol from the liver to bile [97]. They also inhibit fatty acid oxidation enzymes, which leads to a decrease in lipid metabolism in cells [42]. The effects of miR-128-1, miR144, and miR-148a are associated with suppression of ATP-binding cassette transporters A1 expression. The combined effect of these miRs is to reduce the outflow of cholesterol into HDL [44,45].

#### 3.2.6. Changes in Low-Density Lipoprotein Cholesterol Homeostasis

Associated with a decrease in HDL levels, miRs (for example, miR-128-1 and miR-148a) contribute to an increase in LDL levels by suppressing the expression of the LDL receptor, through which peripheral cells absorb lipids from circulating LDL. Thus, inhibition of miR-128-1 and miR-148a in mice led to increased expression of LDL receptor and increased the clearance of circulating LDL, which subsequently led to a decrease in LDL levels in the plasma of mice [44].

MiR-30c reduces LDL levels in the blood. It has been established that overexpression of this microRNA in mice reduces the assembly (folding) and secretion of lipoproteins containing apoprotein B by suppressing microsomal triglyceride transporter protein, which ultimately leads to a decrease in LDL levels in the blood plasma [46].

#### 3.2.7. Changes in the Processes of Atherogenesis

Atherogenesis is an important domain of the pathogenesis of MetS and AIMetS. It has been shown that long-term use of first-generation ASMs (valproic acid, phenytoin, carbamazepine, and phenobarbital) can increase the risk of atherosclerosis and associated cardiovascular diseases in patients with epilepsy [91]. In recent years, it has been demonstrated that miRs, in addition to the previously described mechanisms of influence on lipid metabolism and adipogenesis, regulate the processes of atherogenesis. Thus, inhibition of miR-33 increases cholesterol transport from macrophages to plasma, liver, and feces by more than 80%, which can prevent foam cell formation and atherosclerosis by increasing ATP-binding cassette transporters A1 activity and, as a result, increasing HDL levels. The hypo-expression of miR-33 is also interrelated with other atheroprotective effects, including regulation of functional polarization of macrophages [42].

MiR-144 is a strong inhibitor of cholesterol outflow from various tissues, including macrophages. Hypo-expression of miR-144 in animal models slows down the progression of atherosclerosis [45], and hyperexpression of miR-30c reduces the development of atherosclerosis by inhibiting LDL biogenesis, which was confirmed in mouse models with *ApoE*-mutants and *Ldlr*-mutants [46].

#### 3.2.8. The Development of Fatty Hepatosis (Fatty Liver Disease)

NAFLD is the most common cause of chronic liver disease in the world and one of the many manifestations of insulin resistance and diabetes. NAFLD is a progressive disease characterized by liver insulin resistance and inflammation associated with fat accumulation in the liver. For example, patients taking valproic acid for a long time have an increased risk of developing NAFLD [98]. MiR-27a suppresses the progression of NAFLD in mouse models by reducing the activity of lipid biosynthesis enzymes FAS and sterol-CoA desaturase 1. In contrast to NAFLD, alcohol-associated fatty liver disease shows a marked decrease in miR-122 expression [42].

In contrast, hyperexpression of miR-34a (in both humans and mice) promotes the development of NAFLD. Inhibition of this miR in a mouse model had a therapeutic effect in NAFLD, which was associated with miR-34a-mediated repression of PPARα and subsequent suppression of fatty acid metabolism in the liver [42].

#### 3.2.9. Changes in Insulin Responsiveness

It is known that long-term reception of older-generation ASMs (valproic acid and phenytoin) can have a negative effect on insulin receptors and on various insulin signaling pathways. As a result, the risk of developing insulin resistance as one of the leading domains of AIMetS increases in adults and children with epilepsy and epileptic syndromes [13,14,99,100,101].

Some miRs reduce the responsiveness of cells of various organs and tissues to insulin. The highly conserved microRNA let-7 is expressed in skeletal muscles, where it inhibits the type 2 insulin receptor and the insulin-like growth factor receptor 1, thereby reducing the responsiveness of muscle tissue to insulin [42].

Hyperexpression of miR-15b blocks IRS1 insulin receptors in hepatocytes, contributing to the development of hepatic insulin resistance [42].

MiR-19 inhibits the lipid homologue of phosphatase and tensin involved in insulin signaling in the liver [42,102].

The miR-33 family blocks the type 2 insulin receptor substrate, which is a key component of the insulin signaling pathway [42].

In adipocytes, miR103 and miR-107 suppress caveolin expression, which leads to a weakening of the strength of signals transmitted to insulin receptors [47].

Hyperexpression of miR-143 causes insulin resistance due to inhibition of the protein bound to oxysterol and block of activation of the PI3K/AKT pathway by insulin [42,103].

Circulating miR-155 secreted by macrophages inhibits insulin signaling by suppressing PPARγ expression in myocytes (skeletal muscles and myocardium) and hepatocytes [17,42].

Genes involved in the regulation of glucose metabolism (*IRS1* and *SLC2A4*) are targets for miR-223. Also, this miR modulates the expression of the insulin-regulated glucose transporter protein type 4. Dysregulation of the expression of this miR can suppress the insulin signaling cascade, which can lead to insulin resistance and type 2 diabetes [39].

MiR-378 suppresses the expression of p110a, the catalytic subunit of PI3K. Since PI3K is a key transducer of insulin signaling, sustained hyperexpression of miR-378 leads to insulin resistance [48], and miR-802 affects the expression of the HNF1B transcription factor, enhancing the regulation of HNF1B-associated *Socs1* and *Socs3* genes, which impairs insulin signal transmission by inhibiting phosphorylation of IRS proteins [49].

#### 3.2.10. Changes in Insulin Expression and Secretion by B Cells of Pancreatic Langerhans Islets

Older-generation ASMs (valproic acid and phenytoin) are associated with a greater risk of decreased insulin expression compared to new-generation ASMs (levetiracetam) [14,104]. This mechanism is based on the regulation of glucose transporter protein type 4 expression by PPARγ. However, valproic acid has a dose-dependent effect, increasing the expression of PPARγ at therapeutic doses in patients with epilepsy [17].

The expression of insulin as well as the fusion and release of insulin granules is controlled by miRs.

MiR-7a inhibits the transcription of the insulin gene by suppressing the expression of Pax6 and also inhibits the transcription of the gene encoding the insulin-like peptide Ilp2 by an unknown mechanism. In addition to regulating insulin expression, miR-7a reduces the amount of insulin released by beta cells of pancreatic Langerhans cells by suppressing proteins involved in cytoskeleton remodeling. This miR suppresses the expression of homologous genes (*Cpa* and *Capza1*) necessary for the release of insulin-like peptides and an increase of their level in the blood. The proteins encoded by these genes bind to the pointed ends of F-actin and prevent polymerization and depolymerization of actin filaments, inhibiting the active growth of actin filaments and stabilizing the cytoskeleton. Hyperexpression of this microRNA leads to inhibition of the production of alpha-synuclein, which acts as a molecular chaperone in the assembly of protein complexes associated with synaptosomes (SNARE complexes). In turn, alpha-synuclein and SNARE fuse intracellular transport vesicles with the target membrane, limiting SNARE-dependent assembly of the insulin and merger of insulin granules [42].

MiR-26a is expressed in beta cells of the islets of Langerhans of the pancreas and inhibits the expression of voltage-dependent subunit-C of L-type calcium channels α1, which mediates the influx of calcium and SNARE-dependent fusion of insulin granules with the plasma membrane of beta cells and the subsequent release of insulin into the systemic circulation [50].

The production of monocarboxylate transporter 1 in beta cells of pancreatic Langerhans islets can be inhibited by hyperexpression of three miR-29 paralogs, which directly suppress the matrix RNA of the *MCT1* gene. This transporter protein transports lactate and pyruvate into the mitochondria. When monocarboxylate transporter 1 production is inhibited, beta cells stop producing insulin during physical activity, which leads to a significant increase in circulating lactate and pyruvate levels in the systemic bloodstream [42].

MiR-130a, miR-130b, and miR-152 reduce levels of glucokinase and pyruvate dehydrogenase subunit E1-a1, which converts pyruvate produced by glycolysis into acetyl-CoA in mitochondria. Glucokinase acts at the first stage of glycolysis. Hyperexpression of miR-130a, miR-130b, and miR-152 reduces the intracellular ATP/ADP ratio and insulin synthesis and secretion [51].

Experimental hyperexpression of miR-187 in an animal model led to inhibition of homeodomain interacting protein kinase, weakening glucose-induced insulin secretion in vitro [42], and experimental hyperexpression of miR-200 (in an animal model) led to apoptosis of beta cells of the islets of Langerhans by suppressing the expression of the anti-apoptosis and stress resistance gene *dnajc3* and the apoptotic caspase inhibitor gene *xiap* [54].

MiR-204 suppresses the expression of glucagon-like peptide type 1 receptor on the surface of beta cells of the pancreatic islets of Langerhans, thereby blocking glucagon-like peptide-induced insulin release from beta cells by increasing their responsiveness to glucose [42].

MiR-375 works by a mechanism similar to miR-7, affecting the expression of myotrophin, which binds to free CAPZA1 and regulates its activity, preventing CAPZA1 from binding to the sharp end of F-actin [42].

MiR-802 reduces insulin expression by blocking the binding of the promoter of the insulin-encoding gene to NEUROD1, a key activator of insulin transcription. Inhibition of insulin secretion works according to a similar principle to miR-26a, interfering with calcium influx by suppressing the WNT5A receptor FZD5, which transmits signals through Ca2+/calmodulin-dependent protein kinase II, stimulating the activity of the high-voltage calcium channel for calcium influx [53].

In contrast, several other microRNAs (miR-24, miR-26, miR-30d, miR-148, and miR-182) serve as activators of insulin expression in beta cells by inhibiting insulin transcription repressors.

Animal model studies have demonstrated that miR-26, miR-148, miR-182, and miR-24 increase the expression of matrix RNA of insulin in beta cells of Langerhans by inhibiting insulin transcription repressor genes *Sox6* (miR-24 and miR-148a) and *Bhlhe22* (miR-26 and miR-182) [54]. When miR-30d expression is completely blocked, the expression of the insulin gene induced by an increase in glucose levels stops, proving that miR-30d is a key mediator of glucose signaling in beta cells of the islets of Langerhans of the pancreas [42].

#### 3.2.11. Changes in Glucose Metabolism

MiR-19a regulates the process of glycogenesis, suppressing it by inhibiting the expression of the lipid homologue of phosphatase and tensin in hepatocytes. It is known that this protein indirectly activates the enzyme glycogen synthase kinase type 3, which inhibits glycogen synthase responsible for glycogenesis [42]. Hyperexpression of miR-33 suppresses the functional activity of two key glycogenolytic enzymes—glycogen phosphorylase and phosphoglucomutase—which leads to accumulation of glycogen in the liver [42], and hyperexpression of miR-27a suppresses the functional activity of glycogen phosphorylase, phosphoglucomutase, and α-glucosidase, leading to glycogen accumulation in skeletal muscles and myocardium [42]. MiR24, miR-26, miR-30d, miR-148, and miR-182 indirectly enhance glycogenesis by activating insulin expression [42,54].

MiR-27, miR-29, and miR-451-1 inhibit liver glycogenesis by regulating the functional activity of the gluconeogenic pathway (glycerol kinase and transcription factor Forkhead Box Protein O1) [42]. MiR-33b directly suppresses the expression of two key gluconeogenesis genes encoding phosphoenolpyruvate carboxylase 1 and glucose-6-phosphatase, which suppresses gluconeogenesis [42]. MiR-466b directly inhibits the expression of phosphoenolpyruvate carboxykinase, a key gluconeogenetic enzyme [42].

The mechanisms of suppression of glucose metabolism in other miRs have been described previously [42,47,48,49,50,51,52,53].

#### 3.2.12. Changes in Appetite Regulation

By affecting various structures of the brain as well as many of the signaling pathways described below, ASMs are able to affect appetite regulation and cause an eating disorder associated with AIMetS [105].

In the course of the studies, it was found that microRNAs are involved in the regulation of appetite by targeting the proopiomelanocortin neurons and neuropeptide Y (NPY) in the arcuate nuclei of the hypothalamus.

MiR-7a is highly expressed in NPY/AgRP neurons, which have an anorexic effect [60].

Overexpression of miR-200a in the hypothalamus is associated with impaired food consumption control and impaired leptin/insulin signaling through changes in expression of type 2 insulin receptor. By a similar mechanism, miR-141 and miR-429 have an anorexic effect [57]. Starvation leads to hyperexpression of let-7a, miR-9, mar-30e, miR-132, miR-145, and miR-218, which indicates the orexigenic effect of these miRs [59].

With a decrease in leptin production, the expression levels of miR-383, miR-384-3p, and miR-488 increase, which leads to inhibition of the messenger RNA of the *POMC* gene encoding a key anorexic regulator of food consumption [57]. MiR-342 is associated with an increased population and increased activation of NPU-orexigenic neurons, which leads to increased food consumption [58] and an increased risk of developing central obesity.

Appetite-suppressing miRs include miR-33 and miR-103. Changes in eating behavior characterized by appetite suppression can be mediated by increasing the expression of NPY and inhibition of proopiomelanocortin neurons in the hypothalamus as well as by suppressing the activity of agouti-related peptide neurons (AgRP neurons) in the hypothalamus as a result of hyperexpression of miR-33 [55]. In addition, miR-103 hyperexpression is known to inhibit the phosphatidylinositol-3 kinase/rapamycin target signaling pathway in mammals (PI3K)/AKT/mTOR), which leads to appetite suppression and a reduced risk of obesity due to hyperphagia [57].

#### 3.2.13. Changes in the Expression of Neuropeptide Y

Long-term administration of certain ASMs (for example, valproic acid and topiramate) can lead to an increase in the level of NPY and other biomarkers of AIMetS in the serum [13,106].

MiRs that reduce NPY expression include let 7b, miR29b, miR-33, miR-140-miR-143, and miR-503. Let-7b and miR-143 bind to the *Npy* 3’ UTR and repress the *Npy* mRNA, which was confirmed by luciferase analysis in an experiment using an animal model [61]. The mechanism of action of miR-33 on NPY expression in AgRP neurons was described above [55]. Hyperexpression of miR-29b inhibits *Npy* mRNA in the mHypoE-46 cell line [61]. MiR-140 causes a decrease in NPY levels in clonal hypothalamic cell lines mHypoE-41. Also, miR-503 reduces NPY expression by lowering *Npy* mRNA levels [61].

In addition, miRs associated with an increase in NPY expression have been identified. Thus, hyperexpression of miR-708 and miR-2137 in hypothalamic cell models increases the level of *Npy* mRNA expression through an indirect mechanism that does not involve direct binding to *Npy* 3’UTR [61].

#### 3.2.14. Changes in Leptin Responsiveness

In recent years, the effect of new generation ASMs on changes in serum levels of the protein hormone leptin energy metabolism and adipocyte responsiveness to leptin has been actively discussed. This hormone, by affecting on cellular receptors in the arcuate and ventromedial nuclei of the hypothalamus, participates in appetite regulation and prevents hyperphagia and the development of obesity. Changes in blood leptin levels can be considered as a perspective biomarker of AIMetS in patients with epilepsy and epileptic syndromes [16,106,107].

An increase in hypothalamic responsiveness to leptin may be mediated by the anorexic effect of let-7a, miR-9, miR-30e, miR-132, miR-145, miR-218, and miR-342 [58,59,60]. On the contrary, hyperexpression of miR-15a, miR-16, miR-33, miR-200a, miR-200b, miR-223, miR-363, miR-429, and mil-532 leads to a decrease in cell responsiveness to leptin.

A negative correlation has been shown between an increase in circulating levels of miR-15a, miR-16, miR-223, miR-363, and miR-532 and a decrease in plasma leptin levels [39]. In addition, hyperexpression of miR-200a, miR-200b, and miR-429 was detected in orexigenic hypothalamic neurons in knockout mice with primary leptin deficiency and primary leptin receptor deficiency. MiR-200a directly inhibits type 2 insulin receptor and leptin receptor, which are its direct targets. Inhibition of miR-200a in the hypothalamus increases the expression of *LepR* and *Irs2* mRNAs, increasing the responsiveness of orexigenic hypothalamic neurons to leptin and insulin [60].

#### 3.2.15. Changes in Orexin Expression

The development of AIMetS in children and adults with epilepsy and epileptic syndromes may be directly related to the effect of ASMs on molecules involved in the regulation of systemic metabolism, in particular on the neuropeptide orexin synthesized by hypothalamic neurons [108]. Some miRs inhibit orexin expression, which is of clinical interest since this neuropeptide is not only involved in the regulation of sleep and wakefulness cycles, autonomic functions but also in the regulation of eating behavior.

In particular, miR-137, miR-637, miR-654, and miR-665 inhibit *Hcrt* mRNA in hypothalamic neurons [50].

#### 3.2.16. Changes in Testosterone Expression

Some ASMs of the first and new generations can also negatively affect testosterone expression [109]. Experimentally, it was demonstrated that the introduction of miR-320 into the mouse ovary in vivo led to a significant increase in testosterone production. On the contrary, hyperexpression of miR-320 reduces the synthesis of 17-estradiol and the proliferation of granulosa cells of the ovaries by inhibiting estradiol factor 1 and steroidogenesis factor-1 [63].

Transfection of primary cells of the human ovarian granulosa by pre-miR-15a leads to a decrease in the level of mitogen-activated protein kinase/ERK1,2 and caspase 3 proteins associated with proliferation and apoptosis and stimulates testosterone secretion [63]. Inhibition of testosterone expression is associated with hyperexpression of miR-150, which, affecting on the acute steroid regulatory protein, negatively regulates testosterone production in mouse Leydig cells both in vivo and in vitro [63].

#### 3.2.17. Changes in the Expression of Thyroid Hormones

It is known that long-term use of ASMs, especially of the first generation, is associated with changes in the expression of thyroid hormones and impaired thyroid function. Previous studies have confirmed an ASMs-induced increase in thyroid-stimulating hormone expression in patients with epilepsy [109,110].

A decrease in thyroid hormone levels strongly correlates with increased concentrations of total cholesterol, LDL, and TG in the serum. At the same time, the pathogenetic progression of dyslipidemia associated with secondary hypothyroidism may correlate with a decrease in serum concentration of thyroid hormones and an increase in serum concentration of thyroid-stimulating hormone. Thus, this indicates that ASM-induced hypothyroidism can cause dyslipidemia and related metabolic disorders, including AIMetS [111].

Several miRs (for example, miR-21, miR-146, and miR-214) that induce the expression of thyroid hormones have been identified. MiRs may control the expression of thyroid hormones by regulating deiodinase enzymes. The proteins deiodinase 1 and deiodinase 2 catalyze the conversion of T4 to T3 in target tissues, increasing the intracellular level of the active hormone. Deiodinase 3 causes hormone inactivation because it converts T4 and T3 into inactive metabolites (rT3 and T2) by deiodation along the inner ring [56]. Hyperexpression of miR-21, miR-146, and miR-214 leads to inhibition of *Dio3* mRNA in an animal model experiment, thereby increasing the level of thyroid hormones [56].

In addition, some miRs have been shown to reduce the expression of thyroid hormones [56]. At the same time, miR-27, miR-155, miR-181, miR-200a, miR-221, miR-246, and miR-425 reduce the expression of *Trβ*, which together with the regulation of deiodinase 1 leads to local hypothyroidism and decreased thyroid hormone signaling. Hyperexpression of miR-224 and miR-383 repress *Dio1* mRNA, which leads to a decrease in thyroid hormone production [56].

#### 3.2.18. Changes in Parathyroid Hormone Expression

Metabolic disorders associated with vitamin D deficiency act synergistically with other pathways, contributing to weight gain induced by taking ASMs [112]. ASMs can alter the level of parathyroid hormone in the blood and impair vitamin D metabolism and decrease its level [113,114,115].

MiR-27b, miR-136b, miR-146b, and miR-503 increase expression of parathyroid hormone. Also, miR-27b inhibits the expression of the *VDR* gene encoding the nuclear vitamin D receptor, thereby reducing cell responsiveness to vitamin D, contributing to the development of secondary hyperparathyroidism [64]. The calcium-responsive receptor, expressed on the membranes of parathyroid cells, determines the specific responsiveness of parathyroid cells to increased extracellular calcium by inhibiting the release and synthesis of parathyroid hormone through the intracellular inositol triphosphate pathway [108,109]. Some miRs (miR-135b, miR-146b, and miR-503) are suppressors for *Casr* mRNAs that increase expression of parathyroid hormone by inhibiting the calcium-responsive receptor inhibitor [64].

Hyperexpression of miR-24 inhibits production of parathyroid hormone by binding CDKN1B/p27, CDKN2A/p16, TGFβ1, and CASP8 transcripts, which are involved in the development of hyperparathyroidism [64].

## 4. Discussion

Drug-induced MetS in general and AIMetS [13,116] in particular have remained at the forefront of scientific discourse for many years, with a tendency to dynamically review clinical and laboratory biomarkers as our understanding of the mechanisms of their pathogenesis expands. In particular, in 2023, the International Diabetes Federation (IDF) [9] experts developed a personalized approach to assessing waist circumference in adult patients with MetS depending on their ethnicity and race (Table A2). Additional laboratory markers are also offered (Table A3).

In recent decades, a large number of publications have appeared claiming that epigenetic biomarkers, including miRs, will be the next breakthrough in personalized medicine. However, it should be recognized that the number of epigenetic biomarkers that have received widespread clinical recognition and implementation in real clinical practice is not yet large, and even fewer have been approved by the FDA, EMA, Federal Service for Supervision of Health Care and Social Development of the Russian Federation, and the Ministry of Industry and Trade of the Russian Federation.

We analyzed 28 studies on the role of miRs in key mechanisms of MetS and pathogenesis of AIMetS, conducted with the participation of humans (clinical studies n = 6) and animal models (experimental/preclinical studies n = 22). It should be noted that most of the studies conducted in recent decades and analyzed by us were conducted on animal models and cell cultures. Studies involving patients with MetS in general and AIMetS in particular in patients with epilepsy have so far been isolated and performed on small samples, which does not allow us to summarize the presented results and translate them into real clinical practice.

**Figure 4:**
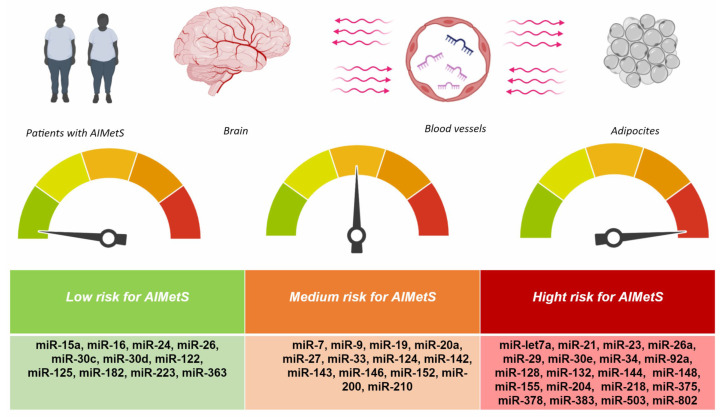
MicroRNAs’ (miRs) gradation based on the risk of antiseizure medication induced metabolic syndrome (AIMetS). Note: Low risk for miRs with protective properties for two or more mechanisms of pathogenesis of AIMetS; medium risk for miRs with predictive properties for one mechanism of the pathogenesis but protective for the other; high risk for miRs with predictors properties of two or more mechanisms of AIMetS pathogenesis.

The responsiveness and specificity of epigenetic biomarkers is low when analyzing one or more (2–3) miRs as biomarkers of AIMetS, and therefore, it is necessary to evaluate the signature of miRs that induce or inhibit several major domains (pathogenetic mechanisms) of AIMetS (Table 2). We analyzed which miRs are associated with more than one pathogenic mechanism of AIMetS. Based on this, we identified miR signatures that can be used to assess the risk of developing AIMetS and its unfavorable course as low, medium, or high (Figure 4).

The introduction of next-generation genetic sequencing platforms (for example, the Illumina platform) and their use for miR sequencing as well as the parallel development of complex computational methods for miR analysis will expand the capabilities of clinical laboratories to use this type of epigenetic biomarkers for predicting and diagnosing AIMetS in children and adults with epilepsy and epileptic syndromes.

The transition to personalized neurology reflects modern approaches to the individual assessment of the safety and risk of pharmacotherapy for epilepsy and epileptic syndromes in children and adults using ASMs as basic and long-term prescribed drugs [117,118].

MicroRNAs have been identified as perspective biomarkers in patients with various forms of epilepsy and are also considered perspective biomarkers for assessing the safety and drug resistance of ASMs [119,120,121].

This review demonstrates the potential role of circulating microRNAs as prognostic or predictive molecular biomarkers of AIMetS, especially those microRNAs that are involved in several links in the pathogenesis of AIMetS (Figure 2), which is of undoubted clinical interest in neurology. The study of these miRs in the future is important for the subsequent planning of bridge and associative studies, ensuring the translation of the results of fundamental research into the practice of a neurologist. Considering that circulating miRs are stable molecules that are stable during sample preparation and repeated freeze–thaw cycles, the study of their level in peripheral blood is promising due to the expected high repeatability of the results obtained in different patients with AIMetS.

This review demonstrates that the role of miRs in regulating such mechanisms of AIMetS pathogenesis as oxidative stress [37,38], systemic inflammation [39,40], adipogenesis and central obesity development [39,40,41,42,43], and glucose and insulin metabolism [42,47,48,49] has been the most studied. These mechanisms are significant because they determine the development of manifestations such as abdominal obesity, hyperglycemia, triglyceridemia, and decreased serum HDL cholesterol. The role of miRs in additional mechanisms of AIMetS pathogenesis is less studied, including testosterone [61], orexin [60], and parathyroid hormone expression [56]. It is worth noting that miRs can be used not only for diagnosis but also for therapy and even as potential therapeutic molecules. One of the ways to affect miRs is through an antagomirs—a small synthetic RNA whose purpose is to block the action of a specific miR in vivo. In their paper, Pamela Agbu et al. [42] noted that the use of anti-miR-33, anti-miR-128-1, and anti-miRs-144 and -148 causes an increase in HDL, followed by a decrease in manifestations of atherosclerosis. These authors suggested that miRs could be used to treat atherosclerosis, with miR-30C being an inhibitor of LDL biogenesis. Therefore, an increase in its expression could have atheroprotective properties. Sarmistha Saha showed that miR-12-3p antagomirs reduce liver damage by increasing levels of Sirt1, NRF2, SIRT3, NQO1, and HO-1 proteins. Kaushik Das et al. [40] mentioned that treatment with an miR-10 inhibitor increases the survival of inflamed chondrocytes. The use of miR mimic drugs and antagomirs is being actively investigated and opens up broad prospects for the treatment and prevention of many diseases, including MetS and AIMetS, by targeting individual mechanisms of pathogenesis. However, the FDA and other regulatory agencies have not yet approved any of these [122].

At the same time, the results of some of the studies we analyzed yielded contradictory results. For example, the pro-inflammatory [40] let-7a was associated with insulin resistance [42] and had orexigenic properties associated with increased responsiveness to leptin [59]. However, its paralog (let-7b) had an inhibitory effect on NPY expression, which led to appetite suppression and a reduced risk of central obesity [61]. MiR-7 induces lipid metabolism [42] and prevents the development of systemic inflammation [40], but it suppresses carbohydrate metabolism, reducing insulin expression [42], and has an orexigenic effect [60]. The anti-inflammatory miR-9 [40] also has an orexigenic effect associated with increased responsiveness to leptin [59]. Anti-inflammatory miR-15a has been associated with decreased responsiveness to leptin [39] and induction of testosterone expression [63]. However, hyperexpression of its paralog (miR-15b) contributed to the development of hepatic insulin resistance [42]. MiR-19 induces glycogenogenesis [42] and is associated with a decrease in oxidative stress [37], but it contributes to the development of insulin resistance [42]. Inhibiting systemic inflammation, miR-20a [40] can be an inducer of adipogenesis [41], but its two paralogs (miR-26 and miR-26a) have fundamentally different effects on glucose metabolism. MiR-26 activates insulin expression and, as a result, enhances carbohydrate metabolism by inhibiting insulin transcription regulators [54], but its paralog (miR-26a) inhibits the SNARE-dependent release of insulin into the systemic circulation [50].

MiR-27 and its paralogs have contradictory effects on the pathogenesis of MetS and AIMetS. Thus, miR-27a inhibits the Nrf2 antioxidant pathway [37] and promotes the development of systemic inflammation [40], as well as leads to the accumulation of glycogen in skeletal muscles and myocardium [42]. However, miR-27a suppresses adipocyte differentiation [41] and regulates lipid metabolism in the liver, preventing the development of NAFLD together with miR-27b [36]. MiR-27 inhibits adipogenesis through activation of the Wnt pathway in 3T3-L1 preadipocytes [41] but at the same time reduces blood glucose production by acting on enzymes of the gluconeogenic pathway [42] and promotes the development of hypothyroidism [56].

The miR-33 family also has a contradictory effect on the pathogenesis of MetS. In general, the hypo-expression of miRs of this family contributes to the development of central obesity and insulin resistance. Paralogs miR-33a and miR-33b coordinate the inhibition of the reverse transport of cholesterol from the periphery back to the liver, inhibit fatty acid oxidation enzymes, and inhibit cholesterol excretion from the body. Inhibition of miR-33a/b has atheroprotective effects, such as regulation of functional polarization of macrophages and increased transport of cholesterol from macrophages to plasma, liver, and feces, which prevents the formation of foam cells and atherosclerosis. The miR-33 family reduces insulin sensitivity, leading to the development of insulin resistance. MiR-33 suppresses glycogenolytic enzymes, which leads to the accumulation of glycogen in the liver. On the contrary, miR-33, miR-33b directly suppresses the expression of gluconeogenesis enzymes [42]. MiR-33 suppresses appetite by reducing the activity of AgRP neurons in the hypothalamus [55] but at the same time reduces responsiveness to leptin [60].

The differences in the presented results may be due to the fact that the fundamental (mainly) and clinical studies we analyzed had a variable design as well as the fact that they did not take into account other modifiable and unmodifiable risk factors for MetS and AIMetS.

## 5. Limitations

The traditional limitation for all miRs is that the complete set of human miRs as well as the functions and mechanisms of individual miRs have not yet been established [123].

The limitation of this review is that studies examining the effect of new-generation ASMs on the development of AIMetS are currently lacking and demonstrate mainly protective properties in relation to the mechanisms of AIMetS pathogenesis [11,18]. Most studies are devoted to the study of AIMetS in patients taking first-generation ASMs such as carbamazepine [15], phenytoin [16], valproic acid [13,15], and phenobarbital [17]. Most of the studies reviewed in this paper were preclinical (n = 22) and were performed on animal models, which is also a limitation since future results of clinical trials in humans may differ from those presented previously. In these preclinical studies, miRs were more frequently detected not in blood but in various tissues and organs because they were carried out on animal models. It should be noted that miRs expressed in tissues may enter the bloodstream or saliva and can be used as biological samples in clinical practice.

## 6. Conclusions

The mechanisms of development and biomarkers of AIMetS are an actual problem of epileptology, which is still far from being resolved. The development of panels (signatures) of epigenetic biomarkers of this widespread ADR may help to increase the safety of pharmacotherapy of epilepsy and epileptic syndromes in children and adults. However, to increase the sensitivity and specificity of circulating miRs in the blood as biomarkers of AIMetS, it is necessary to conduct “bridge” studies in order to replicate the results of preclinical and clinical studies into real clinical practice.

## Figures and Tables

**Table 1:** Criteria for inclusion and exclusion of publications.

Criteria	Inclusion Criteria	Exclusion Criteria
Type of publication	Article, case report, systematic review, meta-analysis, or Cochrane review	Abstract, poster, or thesis
Access to the publication	Full version is available	Full version is not available
Language of publication	English, Russian	Other languages
Database	PubMed, Scopus, Clinical Keys, Oxford Press, Google Scholar, eLibrary	Other databases
Search Depth	2014–2024 (10 years)	Before November 2024

**Table 2:** The role of circulating microRNAs in the pathogenesis of antiseizure medication induced metabolic syndrome.

References	Mechanism of Pathogenesis		Pathophysiological Role of miRs	
			MiRs that Decrease the Risk of Developing a Pathogenetic Mechanism	MiRs that Increase the Risk of Developing a Pathogenetic Mechanism
[37,38]	Oxidative stress		miR-19b, miR-20a, miR-24, miR-99a, miR-125b, miR-141, miR-152, miR-200a, miR-200c, miR-210, miR-221, miR-455, miR-601, miR-626	miR-1, miR-21, miR-23b, miR-27a, miR-28, miR-29, miR-34a, miR-92a, miR-93, miR-101, miR-106b, miR-128, miR-129, miR-140, miR-142, miR-144, miR-146, miR-148, miR-153, miR-155, miR-181c, miR-193b, miR-320, miR-365, miR-375, miR-383, miR-495, miR-503, miR-802
[39,40]	Systemic inflammation		miR-7, miR-9, miR-10a, miR-15a, miR-16, miR-24, miR-31, miR-124, miR-125, miR-126, miR-142, miR-143, miR-146, miR-149, miR-150, miR-210, miR-223, miR-363	miR-21, miR-23a, miR-27a, miR-29a, miR-34a, miR-34c, miR-92a, miR-132, miR-138, miR-155, miR-200, let-7a
[39,41,42,43]	Adipogenesis and development of central obesity		miR-27, miR-27a, miR-30c, miR-33a, miR-33b, miR-130, miR-145, miR-146a, miR-155, miR-181, miR-182, miR-200b, miR-236, miR-363, miR-344, miR-448, miR-4429	miR-17, miR-20a, miR-21, miR-103, miR-128-1, miR-143, miR-144, miR-146b, miR-148a, miR-194, miR-210, miR-322, miR-375, intronic miR-378
[42,56]	Lipid metabolism		miR-30c, miR-33a, miR-33b, miR-34a, miR-128-1, miR-144, miR-148a, miR-223, miR-246b	miR-7, miR-27a, miR-27b, miR-122
[42,44,45]		Level of high- density lipoprotein cholesterol homeostasis	miR-33a, miR-33b, miR-128-1, miR-144, miR-148b	N/D
[44,46]		Level of low- density lipoprotein cholesterol homeostasis	miR-128-1, miR-148a	miR-30c
[42,45,46]		Atherogenesis	miR-30c	miR-33,miR-144
[42]		Fatty hepatosis (fatty liver disease)	miR-27a, miR-122, miR-223	miR-34a
[39,42,47,48,49]		Insulin resistance	N/D	miR-let7 (muscle tissue), miR-15b, miR-19, miR-29, miR-33a/b (liver), miR-103 (adipose tissue), miR-107 (adipose tissue), miR-143, miR-155, miR-223 miR-378 (liver), miR-451-1, miR-802 (liver)
[42,50,51,52,53,54]		Insulin expression and secretion by B cells of pancreatic Langerhans islets	miR-7a, miR-26a, miR-29, miR-124a, miR-130a, miR-130b, miR-152, miR-187, miR-200, miR-204, miR-375, miR-802	miR-24, miR-26, miR-30d, miR-148, miR-182
[42,47,48,49,50,51,52,53,54]		Glucose metabolism	miR-7a, miR-26a, miR-27, miR-29, miR-33b, miR-103, miR-107, miR-124, miR-130a, miR-130b, miR-143, miR-152, miR-155, miR-187, miR-200, miR-204, miR-336, miR-375, miR-378, miR-451-1, miR-466b, miR-802	miR-19, miR-24, miR-26, miR-27a, miR-30d, miR-33, miR-148, miR-182
[55,57,58,59,60]		Appetite	miR-33, miR-103	let-7a, miR-7a, miR-9, miR-30e, miR-100, miR-132, miR-141, miR-145, miR-200a, miR-218, miR-342, miR-383, miR-384-3p, miR-429, miR-488
[55,61]		Neuropeptide Y expression	let7b, miR-29b, miR-33, miR-140- miR-143, miR-503	miR-708, miR-2137
[39,60]		Leptin tolerance	miR-15a, miR-16, miR-33, miR-200a, miR-200b, miR-223, miR-363, miR-429, miR-532	let7a, miR-9, miR-30e, miR-132, miR-145, miR-218, miR-342
[62]		Orexin expression	miR-137, miR-637, miR-654, miR-665	N/D
[63]		Testosterone expression	miR-150	miR-15a, miR-320
[56]		Thyroid hormones expression	miR-27, miR-155, miR-181, miR-200a, miR-221, miR-224, miR-246, miR-383, miR-425	miR-21, miR-146, miR-214
[64]		Parathyroid hormone expression	miR-24	miR-27b, miR-136b, miR-146b, miR-503

Note: miR—microRNA.

## Abbreviations

ASMs	Antiseizure medications
ADRs	Adverse drug reactions
AIMetS	Antiseizure medications-induced metabolic syndrome
RNA	Ribonucleic acids
NFE2L2/Nrf2	Nuclear factor 2, related to erythroid factor 2
Keap1	Kelp-like ECH-associated protein 1
Sirt	Sirtuin
NAD	Nicotinamide adenine dinucleotide
ATP	Adenosine triphosphate
HO-1	Hemoxidase 1
TNF-α	Tumor necrosis factor alpha
IL	Interleukin
TGF-β	Transforming growth factor-β
NF-κB	Nuclear factor kappa-light-chain-enhancer of activated B cells
PI3K	Phosphoinoside kinase type 3
PPARγ	γ-Activated peroxisome proliferator
C/EBP	CCAAT/enhancer-binding protein
LDL	Low-density lipoprotein
NAFLD	Non-alcoholic fatty liver disease
SNARE	Protein complexes associated with synaptosomes
NPY	Neuropeptide Y
IDF	International Diabetes Federation

## Appendix A

**Table A1:** The main and additional laboratory biomarkers of antiseizure medication induced metabolic syndrome in the blood.

Biomarker	Standard	Change at the MetS	The MetS Symptom
Glucose	>100 mg/dL	High	Insulin resistance
Sialic acid	2.0–2.33 mmol/L	High	CHD Systemic inflammation
Uric acid	M: 202.3–416.5 µmol/L F: 142.8–339.2 µmol/L	High	Obesity
Adiponectin	0.6–1.33 g/L	Low	Insulin resistance
Aldosterone	25–315 pg/mL	High	High blood pressure
Chimerin	116–157.5 ng/mL	High	Central obesity Coronary heart disease
Ghrelin	0–100 ng/L	Low	Central obesity
Insulin	2.6–24.9 µU/mL	High	Insulin resistance
Leptin	M: 2–5.6 ng/mL F: 3.7–11.1 ng/mL	High	Insulin resistance Leptin resistance
Omentin	5.82−71ng/mL M: 200–960 ng/mL (18–29 years) M: 252–712 ng/mL (30–39 years) M: 272–784 ng/mL (40–49 years) F: 242–764 ng/mL (15–29 years) F: 236–560 ng/mL (30–37 years) F: 220–600 ng/mL (38–49 years)	Low	Central obesity Endothelial dysfunction Coronary heart disease
Parathyroid hormone	15–65 pg/mL	High	Cardiovascular diseases
Testosterone	M: 8.64–29.0 nmol/L (18–55 years) F: 0.29–1.67 nmol/L (18–55 years)	Low	Central obesity
Thyroid-stimulating hormone	0.27–4.2 µIU/mL	High	Cardiovascular diseases
Total bilirubin	<21 µmol/L	Low	Oxidative stress
A protein that binds fatty acids in adipocytes	<6.2 ng/mL	High	Central obesity Cardiometabolic diseases
C-peptide	1.1–4.4 ng/mL	High	Insulin resistance
Soluble serum ligand CD40	<3.5 ng/mL	High	Systemic inflammation Coronary heart disease
Cystatin C	0.5–1.2 mg/L	High	High blood pressure
Ferritin	M: 20–250 µg/L F: 10–120 µg/L	Contradictory	Oxidative stress
Fibrinogen	1.8–3.5g/L	High	High blood pressure Coronary heart disease
Fibroblast growth factor 21	M: 3.6–1021.4 pg/mL F: 65.3–1209.8 pg/mL	High	Central obesity Atherosclerosis
Monocytic chemotoxic protein-1	4.7–300 pg/mL	High	Coronary heart disease
Plasminogen activator inhibitor-1	5–40 ng/mL	High	Insulin resistance Coronary heart disease
Retinol-binding protein 4	11–40 µg/mL	High	Central obesity Insulin resistance Cardiovascular diseases
Tumor necrosis factor alpha	<8.1 pg/mL	High	Coronary heart disease
Oxidized low-density lipoprotein	26–117 IU/L	High	Oxidative stress Systemic inflammation
Apolipoprotein A1	M: >1.2 g/L F: >1.4 g/L	Low	Insulin resistance Dyslipidemia Central obesity
Apolipoprotein B	0.6–1.33 g/L	High	Insulin resistance Dyslipidemia Central obesity
Free fatty acids	M: 8.3–10.9 ng/mL F: 11.4–13.6 ng/mL	High	Insulin resistance
High-density lipoproteins	0.7–1.7 mmol/L	Low	Insulin resistance
Low-density lipoprotein cholesterol	<2.6 mmol/L	High	Dyslipidemia Central obesity
Triglycerides	<1.7 mmol/L	High	Dyslipidemia Central obesity
Type 1 superoxide dismutase (in red blood cells)	1200–2000 U/g	Low	Oxidative stress Systemic inflammation
Gamma-glutamyltransferase	M: 10–71 U/g F: 6–42 U/g	High	Oxidative stress Systemic inflammation
Lipoprotein-associated phospholipase A	<200 ng/mL	High	Cardiovascular diseases
25-Hydroxyvitamin D	30–100 ng/mL	Low	Cardiovascular diseases
Vitamin E (tocopherol)	5–18 µg/mL	Low	Oxidative stress

Note: M—male; F—female; MetS—metabolic syndrome.

**Table A2:** Ethnic variability of waist circumference in patients with antiseizure medication induced metabolic syndrome ([110], by authors’ modification).

Ethnic Group	Gender	Waist Girth
Europeoids ^1^	Male	≥94 cm
	Female	≥80 cm
People from South Asia ^2^	Male	≥90 cm
	Female	≥80 cm
Chinese	Male	≥90 cm
	Female	≥80 cm
Japanese ^3^	Male	≥90 cm
	Female	≥80 cm
Ethnic groups from South and Central America ^4^	Male	≥90 cm
	Female	≥80 cm
Ethnic groups of the Eastern Mediterranean and the Middle East (Arabs) ^5^	Male	≥94 cm
	Female	≥80 cm

Note: ^1^ In the United States, ATP III values (102 cm in men; 88 cm in women) are likely to continue to be used for clinical purposes. In future epidemiological studies of populations of European origin, prevalence should be determined using both European and North American indicators, which will allow for more accurate comparisons. ^2^ Based on Chinese, Malay, and Asian-Indian populations. ^3^ Initially, other values were proposed for Japanese, but new data confirm the use of the values shown above. ^4^ It is recommended to use the recommendations for South Asians until more specific data become available. ^5^ It is recommended to use the recommendations for Europeoids until more specific data become available.

**Table A3:** Additional biomarkers for antiseizure medication induced metabolic syndrome ([110] by authors’ modification).

Component of MetS	Biomarker (Diagnostic Method)
Improper distribution of body fat (abdominal obesity)	Total body fat distribution (DEXA). Central distribution of fat (CT/MRI). Biomarkers of adipose tissue: leptin, adiponectin (blood test). Liver fat concentration (MRS).
Atherogenic dyslipidemia (besides the high triglyceride levels and low high-density cholesterol lipoproteins in the blood)	Apolipoprotein B or non-high-density cholesterol lipoproteins (blood chemistry). Small particles of low-density cholesterol lipoproteins (blood chemistry).
Insulin resistance (except for increased prandial blood glucose)	Prandial insulin/proinsulin levels (blood test). HOMA-IR—the higher the index, the lower the responsiveness of cells to insulin, that is, the more severe the insulin resistance (blood test). Insulin resistance according to the Bergman minimal model is a mathematical modeling of the glycemic regulation system in patients with diabetes—three ordinary differential equations describing changes in the concentration of glucose and insulin in plasma as well as the elimination of insulin in the body without taking into account external influences (blood test). Increased concentration of FFA on an empty stomach and during meals (blood test). Glucose tolerance test (blood test).
Vascular dysregulation (besides increased blood pressure)	Detection of markers of endothelial dysfunction (blood test). Microalbuminuria (urine test).
Pro-inflammatory status (systemic inflammation)	Elevated levels of highly responsive CRP (blood test). Increased levels of proinflammatory cytokines, including TNF-α, IL-6, etc. (blood test). Decrease in plasma adiponectin levels (blood test).
Prothrombotic status	Fibrinolytic factors, including PAI-1, etc. (blood test). Blood clotting factors, including fibrinogen, etc. (blood test).
Hormonal status	Pituitary–adrenal system (blood test).

Note: CT—computed tomography; CRP—C-reactive protein; DEXA—dual-energy X-ray absorptiometry; FFA—free fatty acids; HOMA-IR—Homeostasis Model Assessment of Insulin Resistance (HOMA-IR = (glucose level (mmol/L) * insulin level (µU/mL))/22.5); IL-6—interleukin 6; MetS—metabolic syndrome; MRI—magnetic resonance imaging; MRS—magnetic resonance spectroscopy; PAI-1—plasminogen activator inhibitor-1; TNF-α—tumor necrosis factor alpha.

## References

[1] Magheru C., Magheru S., Coltau M., Hoza A., Moldovan C., Sachelarie L., Gradinaru I., Hurjui L.L., Marc F., Farcas D.M. (2022). Antiepileptic Drugs and Their Dual Mechanism of Action on Carbonic Anhydrase. J. Clin. Med..

[2] Biso L., Aringhieri S., Carli M., Scarselli M., Longoni B. (2024). Therapeutic Drug Monitoring in Psychiatry: Enhancing Treatment Precision and Patient Outcomes. Pharmaceuticals.

[3] Costa B., Vale N. (2023). Understanding Lamotrigine’s Role in the CNS and Possible Future Evolution. Int. J. Mol. Sci..

[4] Jakovljević D., Nikolić M., Jovanović V., Vidonja Uzelac T., Nikolić-Kokić A., Novaković E., Miljević Č., Milovanović M., Blagojević D. (2024). Influence of Long-Term Anti-Seizure Medications on Redox Parameters in Human Blood. Pharmaceuticals.

[5] Kaushik S., Chopra D., Sharma S., Aneja S. (2019). Adverse Drug Reactions of Anti-Epileptic Drugs in Children with Epilepsy: A Cross-Sectional Study. Curr. Drug Saf..

[6] The IDF Consensus Worldwide Definition of the Metabolic Syndrome. https://idf.org/media/uploads/2023/05/attachments-30.pdf.

[7] Li W., Qiu X., Ma H., Geng Q. (2023). Incidence and Long-Term Specific Mortality Trends of Metabolic Syndrome in the United States. Front. Endocrinol..

[8] Chong K.S., Chang Y.H., Yang C.T., Chou C.K., Ou H.T., Kuo S. (2024). Longitudinal Economic Burden of Incident Complications Among Metabolic Syndrome Populations. Cardiovasc. Diabetol..

[9] Nazish S. (2023). Obesity And Metabolic Syndrome in Patients with Epilepsy, Their Relation with Epilepsy Control. Ann. Afr. Med..

[10] Beyene Kassaw A., Tezera Endale H., Hunie Tesfa K., Derbew Molla M. (2022). Metabolic Syndrome and Its Associated Factors Among Epileptic Patients at Dessie Comprehensive S pecialized Hospital, Northeast Ethiopia; A Hospital-Based Comparative Cross-Sectional Study. PLoS ONE.

[11] Nair S.S., Harikrishnan S., Sarma P.S., Thomas S.V. (2016). Metabolic Syndrome in Young Adults with Epilepsy. Seizure.

[12] Chen B., Choi H., Hirsch L.J., Moeller J., Javed A., Kato K., Legge A., Buchsbaum R., Detyniecki K. (2015). Cosmetic Side Effects of Antiepileptic Drugs in Adults with Epilepsy. Epilepsy Behav..

[13] Shnayder N.A., Grechkina V.V., Trefilova V.V., Efremov I.S., Dontceva E.A., Narodova E.A., Petrova M.M., Soloveva I.A., Tepnadze L.E., Reznichenko P.A. (2023). Valproate-Induced Metabolic Syndrome. Biomedicines.

[14] Tien N., Wu T.-Y., Lin C.-L., Chu F.-Y., Wang C.C.N., Hsu C.Y., Tsai F.-J., Fang Y.-J., Lim Y.-P. (2023). Association of Epilepsy, Anti-Epileptic Drugs (Aeds), and Type 2 Diabetes Mellitus (T2dm): A Population-Based Cohort Retrospective Study, Impact of Aeds on T2dm-Related Molecular Pathway, and Via Peroxisome Proliferator-Activated Receptor γ Transactivation. Front. Endocrinol..

[15] Rakitin A., Kõks S., Haldre S. (2016). Metabolic Syndrome and Anticonvulsants: A Comparative Study of Valproic Acid and Carbamazepine. Seizure.

[16] Meenakshi-Sundaram S., Sankaranarayanan M. (2021). Epilepsy, Phenytoin, and Atherogenic Risk—Current Perspectives. Neurol. India.

[17] Li Y.X., Guo W., Chen R.X., Lv X.R., Li Y. (2024). The Relationships Between Obesity and Epilepsy: A Systematic Review with Meta-Analysis. PLoS ONE.

[18] Dehury S., Patro P., Sahu L., Nayak L., Mallik A.K. (2023). Evaluation of Metabolic Parameters on Use of Newer Antiepileptics Versus Conventional Antiepileptics in Patients of Generalised Tonic-Clonic Seizure: An Observational Study. Cureus.

[19] Stols-Gonçalves D., Tristão L.S., Henneman P., Nieuwdorp M. (2019). Epigenetic Markers and Microbiota/Metabolite-Induced Epigenetic Modifications in the Pathogenesis of Obesity, Metabolic Syndrome, Type 2 Diabetes, and Non-alcoholic Fatty Liver Disease. Curr. Diabetes Rep..

[20] Pant R., Firmal P., Shah V.K., Alam A., Chattopadhyay S. (2021). Epigenetic Regulation of Adipogenesis in Development of Metabolic Syndrome. Front. Cell Dev. Biol..

[21] Bethesda M. (2001). Biomarkers Definitions Working Group. Biomarkers and Surrogate Endpoints: Preferred Definitions and Conceptual Framework. Clin. Pharmacol. Ther..

[22] Califf R.M. (2018). Biomarker Definitions and Their Applications. Exp. Biol. Med..

[23] Srikanthan K., Feyh A., Visweshwar H., Shapiro J.I., Sodhi K. (2016). Systematic Review of Metabolic Syndrome Biomarkers: A Panel for Early Detection, Management, and Risk Stratification in the West Virginian Population. Int. J. Med. Sci..

[24] Cho Y., Lee S.Y. (2022). Useful Biomarkers of Metabolic Syndrome. Int. J. Environ. Res. Public. Health..

[25] Lauschke V.M., Zhou Y., Ingelman-Sundberg M. (2019). Novel Genetic and Epigenetic Factors of Importance for Inter-Individual Differences in Drug Disposition, Response and Toxicity. Pharmacol. Ther..

[26] Reynolds E.H. (2024). Antiepileptic Drugs, Folate One-Carbon Metabolism, Genetics, and Epigenetics: Congenital, Developmental, and Neuropsychological Risks and Antiepileptic Action. Epilepsia.

[27] García-Giménez J.L., Seco-Cervera M., Tollefsbol T.O., Romá-Mateo C., Peiró-Chova L., Lapunzina P., Pallardó F.V. (2017). Epigenetic Biomarkers: Current Strategies and Future Challenges for Their Use in the Clinical Laboratory. Crit. Rev. Clin. Lab. Sci..

[28] Mironova O.I., Berdysheva M.V., Elfimova E.M. (2023). Microrna: A Clinician’s View of the State of the Problem. Part 2. MicroRNA as a Biomarker. Eurasian Heart J..

[29] Dexheimer P.J., Cochella L. (2020). MicroRNAs: From Mechanism to Organism. Front. Cell Dev. Biol..

[30] Pozniak T., Shcharbin D., Bryszewska M. (2022). Circulating microRNAs in Medicine. Int. J. Mol. Sci..

[31] Brandão-Lima P.N., de Carvalho G.B., Payolla T.B., Sarti F.M., Fisberg R.M., Malcomson F.C., Mathers J.C., Rogero M.M. (2023). Circulating microRNAs Showed Specific Responses according to Metabolic Syndrome Components and Sex of Adults from a Population-Based Study. Metabolites.

[32] Solís-Toro D., Mosquera Escudero M., García-Perdomo H.A. (2022). Association Between Circulating microRNAs and the Metabolic Syndrome In Adult Populations: A Systematic Review. Diabetes Metab. Syndr..

[33] Ghafouri-Fard S., Hussen B.M., Abak A., Taheri M., Jalili Khoshnoud R. (2022). Aberrant Expression of miRNAs in Epilepsy. Mol. Biol. Rep..

[34] Soler-Botija C., Gálvez-Montón C., Bayés-Genís A. (2019). Epigenetic Biomarkers in Cardiovascular Diseases. Front. Genet..

[35] Page M.J., McKenzie J.E., Bossuyt P.M., Boutron I., Hoffmann T.C., Mulrow C.D., Shamseer L., Tetzlaff J.M., Akl E.A., Brennan S.E. (2021). The PRISMA 2020 statement: An updated guideline for reporting systematic reviews. BMJ.

[36] O’Brien J., Hayder H., Zayed Y., Peng C. (2018). Overview of microRNA Biogenesis, Mechanisms of Actions, and Circulation. Front. Endocrinol..

[37] Saha S. (2024). Role of microRNA in Oxidative Stress. Stresses.

[38] Włodarski A., Strycharz J., Wróblewski A., Kasznicki J., Drzewoski J., Śliwińska A. (2020). The Role of microRNAs in Metabolic Syndrome-Related Oxidative Stress. Int. J. Mol. Sci..

[39] Carvalho G.B., Brandão-Lima P.N., Payolla T.B., Lucena S.E.F., Sarti F.M., Fisberg R.M., Rogero M.M. (2023). Circulating miRNAs Are Associated with Low-grade Systemic Inflammation and Leptin Levels in Older Adults. Inflammation.

[40] Das K., Rao L.V.M. (2022). The Role of microRNAs in Inflammation. Int. J. Mol. Sci..

[41] Engin A.B., Engin A. (2022). Adipogenesis-related microRNAs in Obesity. ExRNA.

[42] Agbu P., Carthew R.W. (2021). MicroRNA-mediated Regulation of Glucose and Lipid Metabolism. Nat. Rev. Mol. Cell Biol..

[43] Dong M., Ye Y., Chen Z., Xiao T., Liu W., Hu F. (2020). MicroRNA 182 is a Novel Negative Regulator of Adipogenesis by Targeting CCAAT/Enhancer-Binding Protein α. Obesity.

[44] Wagschal A., Najafi-Shoushtari S.H., Wang L., Goedeke L., Sinha S., deLemos A.S., Black J.C., Ramírez C.M., Li Y., Tewhey R. (2015). Genome-wide Identification of microRNAs Regulating Cholesterol and Triglyceride Homeostasis. Nat. Med..

[45] Cheng J., Cheng A., Clifford B.L., Wu X., Hedin U., Maegdefessel L., Pamir N., Sallam T., Tarling E.J., de Aguiar Vallim T.Q. (2020). MicroRNA-144 Silencing Protects Against Atherosclerosis in Male, but Not Female Mice. Arterioscler. Thromb. Vasc. Biol..

[46] Irani S., Iqbal J., Antoni W.J., Ijaz L., Hussain M.M. (2018). MicroRNA-30c Reduces Plasma Cholesterol in Homozygous Familial Hypercholesterolemic and Type 2 Diabetic Mouse Models. J. Lipid Res..

[47] Trajkovski M., Hausser J., Soutschek J., Bhat B., Akin A., Zavolan M., Heim M.H., Stoffel M. (2011). MicroRNAs 103 and 107 Regulate Insulin Sensitivity. Nature.

[48] Liu W., Cao H., Ye C., Chang C., Lu M., Jing Y., Zhang D., Yao X., Duan Z., Xia H. (2014). Hepatic miR-378 Targets P110α and Controls Glucose and Lipid Homeostasis by Modulating Hepatic Insulin Signalling. Nat. Commun..

[49] Kornfeld J.W., Baitzel C., Könner A.C., Kornfeld J.W., Baitzel C., Könner A.C., Nicholls H.T., Vogt M.C., Herrmanns K., Scheja L. (2013). Obesity-induced Overexpression of miR-802 Impairs Glucose Metabolism through Silencing of Hnf1b. Nature.

[50] Xu H., Du X., Xu J., Zhang Y., Tian Y., Liu G., Wang X., Ma M., Du W., Liu Y. (2020). Pancreatic β Cell microRNA-26a Alleviates Type 2 Diabetes by Improving Peripheral Insulin Sensitivity and Preserving β Cell Function. PLoS Biol..

[51] Ofori J.K., Salunkhe V.A., Bagge A., Vishnu N., Nagao M., Mulder H., Wollheim C.B., Eliasson L., Esguerra J.L. (2017). Elevated miR-130a/miR130b/miR-152 Expression Reduces Intracellular ATP Levels in the Pancreatic Beta Cell. Sci. Rep..

[52] Belgardt B.F., Ahmed K., Spranger M., Latreille M., Denzler R., Kondratiuk N., von Meyenn F., Villena F.N., Herrmanns K., Bosco D. (2015). The microRNA-200 Family Regulates Pancreatic Beta Cell Survival in Type 2 Diabetes. Nat. Med..

[53] Zhang F., Ma D., Zhao W., Wang D., Liu T., Liu Y., Yang Y., Liu Y., Mu J., Li B. (2020). Obesity-induced overexpression of miR-802 Impairs Insulin Transcription and Secretion. Nat. Commun..

[54] Melkman-Zehavi T., Oren R., Kredo-Russo S., Shapira T., Mandelbaum A.D., Rivkin N., Nir T., Lennox K.A., Behlke M.A., Dor Y. (2011). MiRNAs Control Insulin Content in Pancreatic β-Cells Via Downregulation of Transcriptional Repressors. EMBO J..

[55] Price N.L., Fernández-Tussy P., Varela L., Cardelo M.P., Shanabrough M., Aryal B., de Cabo R., Suárez Y., Horvath T.L., Fernández-Hernando C. (2024). MicroRNA-33 Controls Hunger Signaling in Hypothalamic AgRP Neurons. Nat. Commun..

[56] Aranda A. (2021). MicroRNAs and Thyroid Hormone Action. Mol. Cell. Endocrinol..

[57] Taouis M. (2016). MicroRNAs in the Hypothalamus. Best. Pract. Res. Clin. Endocrinol. Metab..

[58] Zhang D., Yamaguchi S., Zhang X., Yang B., Kurooka N., Sugawara R., Albuayjan H.H.H., Nakatsuka A., Eguchi J., Hiyama T.Y. (2021). Upregulation of *Mir342* in Diet-Induced Obesity Mouse and the Hypothalamic Appetite Control. Front. Endocrinol..

[59] Sangiao-Alvarellos S., Pena-Bello L., Manfredi-Lozano M., Tena-Sempere M., Cordido F. (2014). Perturbation of Hypothalamic Microrna Expression Patterns in Male Rats after Metabolic Distress: Impact of Obesity and Conditions of Negative Energy Balance. Endocrinology.

[60] Derghal A., Djelloul M., Azzarelli M., Degonon S., Tourniaire F., Landrier J.F., Mounien L. (2018). MicroRNAs are Involved in the Hypothalamic Leptin Sensitivity. Epigenetics.

[61] Mak K.W.Y., He W., Loganathan N., Belsham D.D. (2023). Bisphenol A Alters the Levels of miRNAs That Directly and/or Indirectly Target Neuropeptide Y in Murine Hypothalamic Neurons. Genes.

[62] Holm A., Possovre M.L., Bandarabadi M., Moseholm K.F., Justinussen J.L., Bozic I., Lemcke R., Arribat Y., Amati F., Silahtaroglu A. (2022). The Evolutionarily Conserved miRNA-137 Targets the Neuropeptide Hypocretin/Orexin and Modulates the Wake to Sleep Ratio. Proc. Natl. Acad. Sci. USA.

[63] Azhar S., Dong D., Shen W.J., Hu Z., Kraemer F.B. (2020). The Role of miRNAs in Regulating Adrenal and Gonadal Steroidogenesis. J. Mol. Endocrinol..

[64] Vaira V., Verdelli C., Forno I., Corbetta S. (2017). MicroRNAs in Parathyroid Physiopathology. Mol. Cell. Endocrinol..

[65] Martinc B., Grabnar I., Milosheska D., Lorber B., Vovk T. (2024). A Cross-Sectional Study Comparing Oxidative Stress in Patients with Epilepsy Treated with Old and New Generation Antiseizure Medications. Medicina.

[66] Swathi B., Aruna D. (2022). Evaluation of Antioxidant Effects of Antiepileptic Drugs in Adult Epileptic Patients: An Open Label, Non Randomised Interventional Study. J. Clin. Diagn. Res..

[67] Morimoto M., Satomura S., Hashimoto T., Kyotani S. (2017). A Study of oxidative Stress and the Newer Antiepileptic Drugs in Epilepsy Associated with Severe Motor and Intellectual Disabilities. J. Chin. Med. Assoc..

[68] Tkachev V.O., Menshchikova E.B., Zenkov N.K. (2011). Mechanism of the NRF2/KEAP1/ARE Signaling System. Biochemistry.

[69] Zhao M., Li G., Zhao L. (2024). The Role of SIRT1-FXR Signaling Pathway in Valproic Acid Induced Liver Injury: A Quantitative Targeted Metabolomic Evaluation in Epileptic Children. Front. Pharmacol..

[70] Zhang H., Dai S., Yang Y., Wei J., Li X., Luo P., Jiang X. (2024). Role of Sirtuin 3 in Degenerative Diseases of the Central Nervous System. Biomolecules.

[71] Yang W., Nagasawa K., Munch C., Xu Y., Satterstrom K., Jeong S., Hayes S.D., Jedrychowski M.P., Vyas F.S., Zaganjor E. (2016). Mitochondrial Sirtuin Network Reveals Dynamic Sirt3-Dependent Deacetylation in Response to Membrane Depolarization. Cell.

[72] Tang P., Dang H., Huang J., Xu T., Yuan P., Hu J., Sheng J.F. (2018). NADPH Oxidase NOX4 is a Glycolytic Regulator through mROS-HIF1α Axis in Thyroid Carcinomas. Sci. Rep..

[73] Palsamy P., Bidasee K.R., Shinohara T. (2014). Valproic Acid Suppresses Nrf2/Keap1 Dependent Antioxidant Protection through Induction of Endoplasmic Reticulum Stress and Keap1 Promoter DNA Demethylation in Human Lens Epithelial Cells. Exp. Eye Res..

[74] Lin T.K., Chen S.D., Lin K.J., Chuang Y.C. (2020). Seizure-Induced Oxidative Stress in Status Epilepticus: Is Antioxidant Beneficial?. Antioxidants.

[75] Ignacio-Mejía I., Contreras-García I.J., Mendoza-Torreblanca J.G., Medina-Campos O.N., Pedraza-Chaverri J., García-Cruz M.E., Romo-Mancillas A., Gómez-Manzo S., Bandala C., Sánchez-Mendoza M.E. (2023). Evaluation of the Antioxidant Activity of Levetiracetam in a Temporal Lobe Epilepsy Model. Biomedicines.

[76] Basta-Kaim A., Budziszewska B., Lasoń W. (2008). Wpływ Leków Przeciwpadaczkowych na Układ Odpornościowy [Effects of Antiepileptic Drugs on Immune System]. Przegl Lek..

[77] Beghi E., Shorvon S. (2011). Antiepileptic Drugs and the Immune System. Epilepsia.

[78] Dambach H., Hinkerohe D., Prochnow N., Stienen M.N., Moinfar Z., Haase C.G., Hufnagel A., Faustmann P.M. (2014). Glia and Epilepsy: Experimental Investigation of Antiepileptic Drugs in an Astroglia/Microglia Co-Culture Model of Inflammation. Epilepsia.

[79] Godhwani N., Bahna S.L. (2016). Antiepilepsy Drugs and the Immune System. Ann. Allergy Asthma Immunol..

[80] Sun H., Ma D., Cheng Y., Li J., Zhang W., Jiang T., Li Z., Li X., Meng H. (2023). The JAK-STAT Signaling Pathway in Epilepsy. Curr. Neuropharmacol..

[81] Cai M., Lin W. (2022). The Function of NF-Kappa B During Epilepsy, a Potential Therapeutic Target. Front. Neurosci..

[82] Wang Y., Peng J., Bai S., Yu H., He H., Fan C., Hao Y., Guan Y. (2021). A *PIK3R2* Mutation in Familial Temporal Lobe Epilepsy as a Possible Pathogenic Variant. Front. Genet..

[83] Tang X., Chen X., Li X., Cheng H., Gan J., Liu Z. (2022). The TLR4 Mediated Inflammatory Signal Pathway Might Be Involved in Drug Resistance in Drug-Resistant Epileptic Rats. J. Neuroimmunol..

[84] Rafi S.K., Goering J.P., Olm-Shipman A.J., Hipp L.A., Ernst N.J., Wilson N.R., Hall E.G., Gunewardena S., Saadi I. (2021). Anti-Epileptic Drug Topiramate Upregulates TGFβ1 and SOX9 Expression in Primary Embryonic Palatal Mesenchyme Cells: Implications for Teratogenicity. PLoS ONE.

[85] Castañeda-Cabral J.L., Beas-Zárate C., Rocha-Arrieta L.L., Orozco-Suárez S.A., Alonso-Vanegas M., Guevara-Guzmán R., Ureña-Guerrero M.E. (2019). Increased Protein Expression of VEGF-A, VEGF-B, VEGF-C and Their Receptors in the Temporal Neocortex of Pharmacoresistant Temporal Lobe Epilepsy Patients. J. Neuroimmunol..

[86] Cheng L., Xia F., Li Z., Shen C., Yang Z., Hou H., Sun S., Feng Y., Yong X., Tian X. (2023). Structure, Function and Drug Discovery of GPCR Signaling. Mol. Biomed..

[87] Wang N., Han X., Liu H., Zhao T., Li J., Feng Y., Mi X., Zhang Y., Chen Y., Wang X. (2017). Myeloid Differentiation Factor 88 is Up-Regulated in Epileptic Brain and Contributes to Experimental Seizures in Rats. Exp. Neurol..

[88] Poonaki E., Kahlert U.D., Meuth S.G., Gorji A. (2022). The Role of the ZEB1-Neuroinflammation Axis in CNS Disorders. J. Neuroinflamm..

[89] Porretti J., Dalton G.N., Massillo C., Scalise G.D., Farré P.L., Elble R., Gerez E.N., Accialini P., Cabanillas A.M., Gardner K. (2018). CLCA2 Epigenetic Regulation by CTBP1, HDACs, ZEB1, EP300 and miR-196b-5p Impacts Prostate Cancer Cell Adhesion and EMT in Metabolic Syndrome Disease. Int. J. Cancer.

[90] Bahmad H.F., Daouk R., Azar J., Sapudom J., Teo J.C.M., Abou-Kheir W., Al-Sayegh M. (2020). Modeling Adipogenesis: Current and Future Perspective. Cells.

[91] Ghesmati Z., Rashid M., Fayezi S., Gieseler F., Alizadeh E., Darabi M. (2024). An Update on the Secretory Functions Of Brown, White, and Beige Adipose Tissue: Towards Therapeutic Applications. Rev. Endocr. Metab. Disord..

[92] Im D.U., Kim S.C., Chau G.C., Um S.H. (2019). Carbamazepine Enhances Adipogenesis by Inhibiting Wnt/β-catenin Expression. Cells.

[93] Huang C.J., Choo K.B., Chen C.F. (2022). The microRNA-Signaling-Peroxisome Proliferator-Activated Receptor Gamma Connection in the Modulation of Adipogenesis: Bioinformatics Projection on Chicken. Poult. Sci..

[94] Okada S., Nishina M., Koizumi K., Katayama M., Inoue S., Suga S. (2020). Impact of Enzyme-Inducing Anti-Epilepsy Drugs on Lipid Levels in Elderly Patients with Epilepsy. Epilepsy Res..

[95] Muller A.L., Diaz-Arias L., Cervenka M.C., McDonald T.J.W. (2023). The Effect of Anti-Seizure Medications on Lipid Values in Adults with Epilepsy. Epilepsy Behav..

[96] Knebel B., Hartwig S., Jacob S., Kettel U., Schiller M., Passlack W., Koellmer C., Lehr S., Müller-Wieland D., Kotzka J. (2018). Inactivation of SREBP-1a Phosphorylation Prevents Fatty Liver Disease in Mice: Identification of Related Signaling Pathways by Gene Expression Profiles in Liver and Proteomes of Peroxisomes. Int. J. Mol. Sci..

[97] Babu B.S., Varghese C.P., Gilvaz P.C. (2023). Effects of Long Term Antiseizure Medications on Atherosclerosis. J. Med. Sci. Res..

[98] Farinelli E., Giampaoli D., Cenciarini A., Cercado E., Verrotti A. (2015). Valproic Acid and Nonalcoholic Fatty Liver Disease: A Possible Association?. World J. Hepatol..

[99] Rehman T., Sachan D., Chitkara A. (2017). Serum Insulin and Leptin Levels in Children with Epilepsy on Valproate-associated Obesity. J. Pediatr. Neurosci..

[100] Shlobin N.A., Sander J.W. (2020). Drivers for the Comorbidity of Type 2 Diabetes Mellitus and Epilepsy: A Scoping Review. Epilepsy Behav..

[101] Marcovecchio M.L., Petrosino M.I., Chiarelli F. (2015). Diabetes and Epilepsy in Children and Adolescents. Curr. Diabetes Rep..

[102] Li Y.Z., Di Cristofano A., Woo M. (2020). Metabolic Role of PTEN in Insulin Signaling and Resistance. Cold Spring Harb. Perspect. Med..

[103] Ramasubbu K., Devi Rajeswari V. (2023). Impairment of Insulin Signaling Pathway PI3K/Akt/mTOR and Insulin Resistance Induced AGEs on Diabetes Mellitus and Neurodegenerative Diseases: A Perspective Review. Mol. Cell Biochem..

[104] Sarangi S.C., Pattnaik S.S., Dash Y., Tripathi M., Velpandian T. (2024). Is There Any Concern of Insulin Resistance and Metabolic Dysfunctions with Antiseizure Medications? A Prospective Comparative Study of Valproate Vs. Levetiracetam. Seizure.

[105] Buraniqi E., Dabaja H., Wirrell E.C. (2022). Impact of Antiseizure Medications on Appetite and Weight in Children. Paediatr. Drugs.

[106] Sonmez F.M., Zaman D., Aksoy A., Deger O., Aliyazicioglu R., Karaguzel G., Fazlioglu K. (2013). The Effects of Topiramate and Valproate Therapy on Insulin, C-Peptide, Leptin, Neuropeptide Y, Adiponectin, Visfatin, and Resistin Levels in Children with Epilepsy. Seizure.

[107] Shan Y., Chen Y., Gu H., Wang Y., Sun Y. (2023). Regulatory Basis of Adipokines Leptin and Adiponectin in Epilepsy: From Signaling Pathways to Glucose Metabolism. Neurochem. Res..

[108] Arslan G.A., Saygi S., Bodur E., Cicek C., Tezer F.I. (2022). Relation Between Orexin A and Epileptic Seizures. Epilepsy Res..

[109] Burakgazi Dalkilic E. (2021). Effects of Antiepileptic Drugs on Hormones. Neurosci. Lett..

[110] Adhimoolam M., Arulmozhi R. (2016). Effect of Antiepileptic Drug Therapy on Thyroid Hormones Among Adult Epileptic Patients: An Analytical Cross-Sectional Study. J. Res. Pharm. Pract..

[111] Su X., Chen X., Peng H., Song J., Wang B., Wu X. (2022). Novel Insights into the Pathological Development of Dyslipidemia in Patients with Hypothyroidism. Bosn. J. Basic. Med. Sci..

[112] Dontseva E.A., Pilipenko P.I., Shnayder N.A., Petrova M.M., Nasyrova R.F. (2022). Prevalence of Anticonvulsant-Induced Vitamin D Deficiency. Epilepsy Paroxysmal Cond..

[113] Tombini M., Palermo A., Assenza G., Pellegrino G., Benvenga A., Campana C., Naciu A.M., Assenza F., Lazzaro V.D. (2018). Calcium Metabolism Serum Markers in Adult Patients with Epilepsy and the Effect of Vitamin D Supplementation on Seizure Control. Seizure.

[114] Keller A.N., Kufareva I., Josephs T.M., Diao J., Mai V.T., Conigrave A.D., Christopoulos A., Gregory K.J., Leach K. (2018). Identification of Global and Ligand-Specific Calcium Sensing Receptor Activation Mechanisms. Mol. Pharmacol..

[115] Anghel S.A., Dinu-Pirvu C.E., Costache M.A., Voiculescu A.M., Ghica M.V., Anuța V., Popa L. (2024). Receptor Pharmacogenomics: Deciphering Genetic Influence on Drug Response. Int. J. Mol. Sci..

[116] Shnayder N.A., Grechkina V.V., Kissin M.Y., Dmitrenko D.V., Nasyrova R.F. (2024). Role of Neuropeptide Y in Development of Valproate-Induced Eating Behaviour Disorder. Epilepsy Paroxysmal Cond..

[117] Neznanov N.G. (2021). A Paradigm Shift To Treat Psychoneurological Disorders. Pers. Psychiatry Neurol..

[118] Ashurov Z.S. (2023). The Evolution of Personalized Psychiatry. Pers. Psychiatry Neurol..

[119] Martinez B., Peplow P.V. (2023). MicroRNAs as Potential Biomarkers in Temporal Lobe Epilepsy and Mesial Temporal Lobe Epilepsy. Neural Regen. Res..

[120] De Benedittis S., Fortunato F., Cava C., Gallivanone F., Iaccino E., Caligiuri M.E., Castiglioni I., Bertoli G., Manna I., Labate A. (2021). Circulating microRNAs as Potential Novel Diagnostic Biomarkers to Predict Drug Resistance in Temporal Lobe Epilepsy: A Pilot Study. Int. J. Mol. Sci..

[121] Rzepka-Migut B., Paprocka J. (2021). Prospects and Limitations Related to the Use of MicroRNA as a Biomarker of Epilepsy in Children: A Systematic Review. Life.

[122] Iacomino G. (2023). miRNAs: The Road from Bench to Bedside. Genes.

[123] Koshiol J., Wang E., Zhao Y., Marincola F., Landi M.T. (2010). Strengths and Limitations of Laboratory Procedures for microRNA Detection. Cancer Epidemiol. Biomark. Prev..

